# Three-Dimensional Digital Model Reconstruction and Seepage Characteristic Analysis of Porous Polyimide

**DOI:** 10.3390/polym18050591

**Published:** 2026-02-27

**Authors:** Zhaoliang Dou, Shuang Li, Wenbin Chen, Ye Yang, Hongjuan Yan, Lina Si, Qianghua Chen, Kang An, Hong Li, Fengbin Liu

**Affiliations:** School of Mechanical and Materials Engineering, North China University of Technology, Beijing 100144, China; ls202511@126.com (S.L.);

**Keywords:** porous polyimide, computed tomography, three-dimensional reconstruction, Lattice Boltzmann method, seepage/percolation characteristics

## Abstract

This study focuses on porous polyimide (PPI) lubricating materials for high-speed aerospace bearings. Based on their real microstructure, three-dimensional digital model reconstruction and mesoscale seepage characteristics were investigated. First, a sequence of two-dimensional slice images of PPI was obtained using micro-focus X-ray computed tomography (CT). Through image filtering, threshold segmentation, and three-dimensional reconstruction, a highly faithful digital model of the pore structure was constructed, and a quantified pore-network model was further extracted. Second, a multiple-relaxation-time lattice Boltzmann model based on the D3Q27 discrete scheme was established, and its accuracy and stability in complex boundaries and pressure-driven flows were verified using classic benchmark cases. Subsequently, the validated numerical model was applied to the reconstructed PPI pore structure to simulate and systematically analyze the single-phase seepage behavior of lubricating oil. The results show that the lubricant seepage exhibits a strong “preferential flow path” effect, with most of the flow transported through a small number of large-size throats. A clear quantitative relationship exists between the microscopic flow field structure—including velocity distribution, flow paths, and pressure gradient—and the pore-topology features, such as throat-size distribution, connectivity, and tortuosity. This verifies the mesoscale mechanism that “structure governs flow.” The complete technical chain established in this work—“real-structure reconstruction–numerical model validation–seepage mechanism analysis”—provides a reliable theoretical and numerical tool for gaining deeper insight into the lubricant transport behavior in porous polyimide and offers guidance for the microstructural design and optimization of this material.

## 1. Introduction

The widespread application of high-speed bearing components in spacecraft and aerospace systems has rendered their long-term operational stability and reliability a key technical indicator [1]. Under prolonged, high-speed, and high-load operating conditions, the performance of the lubrication system directly determines the service life and operational stability of these components, thereby driving a continuous demand for high-performance lubricating materials.

In this context, porous polyimide (PPI) materials overcome the limitations of traditional materials. They possess high specific strength and specific modulus, along with excellent mechanical properties and good self-lubricating performance at elevated temperatures [2]. Due to these unique properties, particularly their favorable frictional performance under vacuum conditions, PPI is widely used in high-speed bearing components within the aerospace sector. The material’s internal structure features abundant pores and interconnected channels, making it an excellent oil reservoir medium [3]. During operation, lubricant is transported to the contact surface under centrifugal force and frictional heating, forming a transfer film. When rotation ceases, the lubricant is drawn back into the pores via capillary action. This cyclic process of oil storage and supply enables continuous lubrication, effectively extending the service life of the mechanism. Furthermore, PPI retainers not only provide lubrication but can also enhance heat dissipation within the bearing through air convection or lubricant flow, reducing frictional heat generated during high-speed bearing operation.

However, in research concerning lubricant supply performance, existing percolation theories have limitations in elucidating microscopic flow mechanisms, making it difficult to reveal fluid flow behavior at the mesoscale [4]. Coupled with the inadequacy of sub-micron experimental observation techniques, a significant knowledge gap persists in the analysis of fluid motion at the mesoscopic level [5]. Consequently, there is a pressing need to employ three-dimensional reconstruction methods for porous media, based on micro-focus X-ray computed tomography (CT) and digital core technology, to construct digital models possessing authentic pore topological features [6]. Subsequently, numerical simulation methods should be leveraged to conduct in-depth investigations into the seepage laws of fluids within microscopic pore structures [7]. The core of this research approach lies in establishing a complete technical chain of “real-structure reconstruction—numerical simulation validation—performance correlation analysis,” which provides a reliable foundation for thoroughly investigating the seepage behavior and transport mechanisms of lubricants in porous polyimide [8].

To this end, this study first employs micro-focus CT scanning technology to obtain a sequence of two-dimensional slice images of the porous polyimide. Dedicated software is then used for image preprocessing and threshold segmentation [9]. Based on the resulting binarized images, a three-dimensional digital model replicating the real pore structure is constructed. Regarding numerical simulation, the study adopts the Lattice Boltzmann Method (LBM). Two classic benchmark cases—lid-driven cavity flow and Poiseuille flow—are used to verify the correctness of the model and its boundary conditions, ensuring its applicability for flow simulation within complex porous media [10].

Building upon this foundation, this paper conducts a detailed study on the single-phase seepage behavior of lubricant within PPI, based on the realistically reconstructed pore geometry and the validated LBM model. The aims of this study are: (1) to analyze the macroscopic transport characteristics of the lubricant; (2) to reveal the microscopic flow field structure, including velocity/pressure distribution and flow paths; and (3) to establish a quantitative correlation mechanism between pore structural characteristics (such as porosity, tortuosity, and connectivity) and macroscopic seepage performance. This systematic mesoscale analysis seeks to elucidate the fundamental laws governing single-phase lubricant flow within real, complex pore networks. It aims to provide direct theoretical guidance and a numerical predictive tool for performance optimization and design of porous polyimide lubricating materials. Simultaneously, the realistic pore structure model and the validated LBM numerical framework constructed in this study also lay a crucial foundation in terms of models, methods, and parameters for subsequent in-depth research. This includes investigating the influence of surface wettability, microscopic pore-wall morphology (roughness), and their coupled effects on lubricant seepage, dynamic transport processes, and even multiphase flow behavior.

## 2. Characterization of Microstructure and 3D Reconstruction of Porous Polyimide

### 2.1. Micro-Focus CT Scanning and Image Acquisition

Accurate construction of the pore scale is fundamental for seepage analysis, as this step directly impacts the accuracy of seepage process simulations and the reliability of experimental results. Consequently, it has garnered widespread attention from scholars both domestically and internationally, leading to extensive research. Currently, mainstream pore-scale reconstruction methods are primarily divided into two categories [11]: The first is physical reconstruction based on imaging tomography, which utilizes microscopic imaging techniques such as CT and SEM to capture the morphology of real pore structures. The second is numerical reconstruction, commonly including methods such as the particle deposition method (simulating particle packing processes to achieve structural reconstruction), the statistical reconstruction method (reproducing structures based on statistical pore characteristics like porosity and pore size distribution), and the quartet structure generation set method (constructing pore networks by controlling skeleton growth parameters).

In specific studies, various scholars have employed different reconstruction methods to accurately characterize pore structures and investigate their seepage behavior. For instance, Han et al. [12] utilized tomographic CT scanning technology to perform three-dimensional reconstruction of the pore microstructure of rock samples and further analyzed their seepage characteristics. Cai et al. [13], on the other hand, constructed a two-dimensional porous media model based on the quartet structure generation set method and combined it with LBM to study seepage mechanisms under different conditions.

To obtain a three-dimensional model that accurately reflects the pore structure of the sample, this study employed an Rmct-4000 CT scanner for image acquisition of a porous polyimide bulk sample. The original shape of the sample approximated an irregular cuboid, with approximate dimensions of 1 mm (length) × 2 mm (width) × 1 mm (height). The scanning process adopted a layer-by-layer approach, with each layer corresponding to a two-dimensional slice, and the scanning area was rectangular. As the pore sizes within the porous polyimide were predominantly concentrated in the micro-nano scale, a relatively high resolution was required during scanning to effectively capture the fine structures. Therefore, the voxel size for this scan was set to 0.30383 μm (isotropic), corresponding to the 300 nm resolution mentioned in the text. A total of 959 consecutive two-dimensional slices were obtained from the scan. The original image pixel matrix was 2048 × 2048, and the field of view was approximately 622 μm × 622 μm (calculated from the pixel matrix and voxel size), covering a representative volume element with statistical significance within the sample. Reconstruction was performed using the Filtered Back Projection (FBP) algorithm, completed by the software provided with the equipment. The key acquisition parameters for this scan are summarized in Table 1.

**Figure 1:**
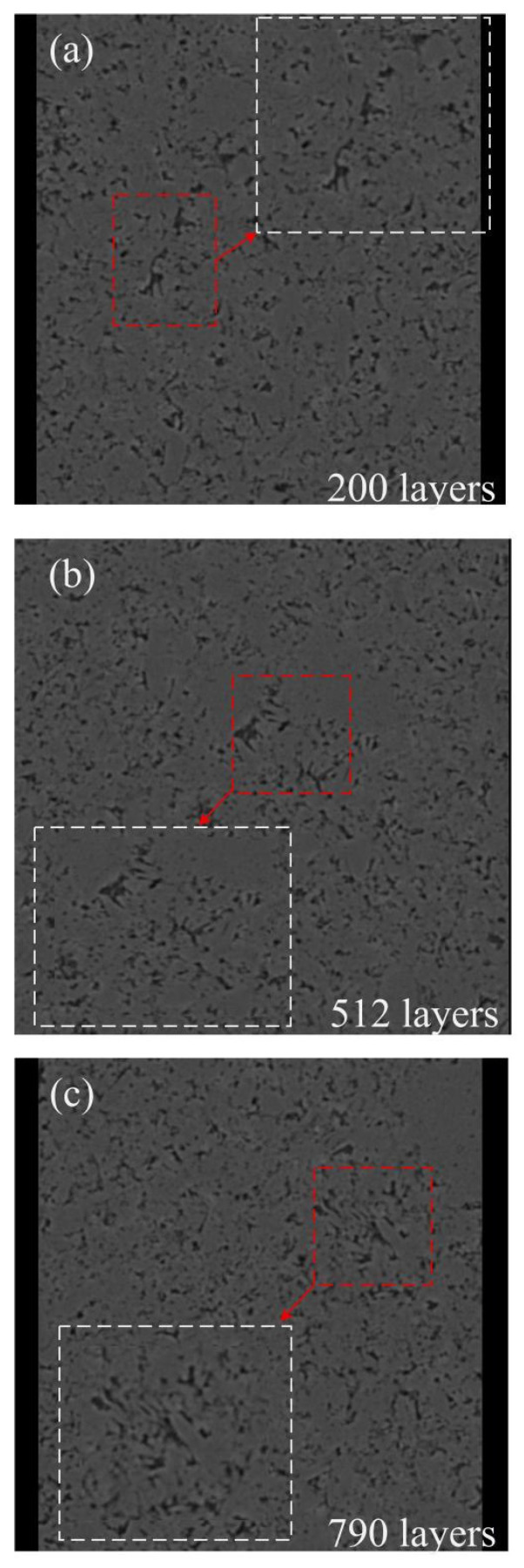
Two-dimensional slice images: (**a**) Slice 150 (near the top of the sample), (**b**) Slice 480 (middle of the sample), and (**c**) Slice 820 (near the bottom of the sample).

Considering the homogeneity of the material’s microstructure (relatively consistent pore distribution and morphology), the scanning results obtained from this single sample are sufficient to reflect the typical pore characteristics of this class of materials. During the image preprocessing stage, to eliminate noise and artifacts at the edges of the scanning field of view, the original slices were cropped using Avizo 2024 software. The blurred edge regions were removed, retaining only the areas with clear internal structures for subsequent three-dimensional reconstruction and porosity analysis. From the preprocessed slice sequence, representative images from three different axial positions—the front, middle, and rear (specifically slice 200, slice 512, and slice 790)—are presented in Figure 1 to visually illustrate the distribution characteristics of the pore structure at different depths within the sample.

Figure 1 presents two-dimensional CT slice images (XY plane) of the porous polyimide at different axial positions. In these images, the black areas represent pores, while the gray areas represent the solid skeleton. The insets in the lower right corners are enlarged views of the corresponding slices (areas within the white dashed boxes), with red arrows pointing to typical pore structures. It can be observed that the pore shapes are irregular, with a size distribution ranging from hundreds of nanometers to several micrometers. Some pores are interconnected, forming network-like channels that provide potential pathways for lubricant transport. Overall, the cropped two-dimensional slice images are clearly imaged with minimal noise and artifacts, demonstrating high image quality. However, to further enhance image clarity and facilitate more accurate identification of pore structures in the subsequent threshold segmentation step, filtering preprocessing is still required to obtain clearer images and a high-quality foundation for analysis.

### 2.2. Image Preprocessing and Pore/Solid Matrix Phase Segmentation

After acquiring the high-resolution micro-focus CT scan sequence of two-dimensional slices, a core task of this study is to bridge the gap from experimental observation to numerical simulation across scales. This involves constructing a three-dimensional digital model from these discrete 2D images that accurately reflects the real internal structure of the porous polyimide. This digital model is not only the foundation for subsequent pore-scale seepage analysis but also serves as the essential geometric prerequisite for conducting numerical simulations using the Lattice Boltzmann Method (LBM).

**Figure 2:**
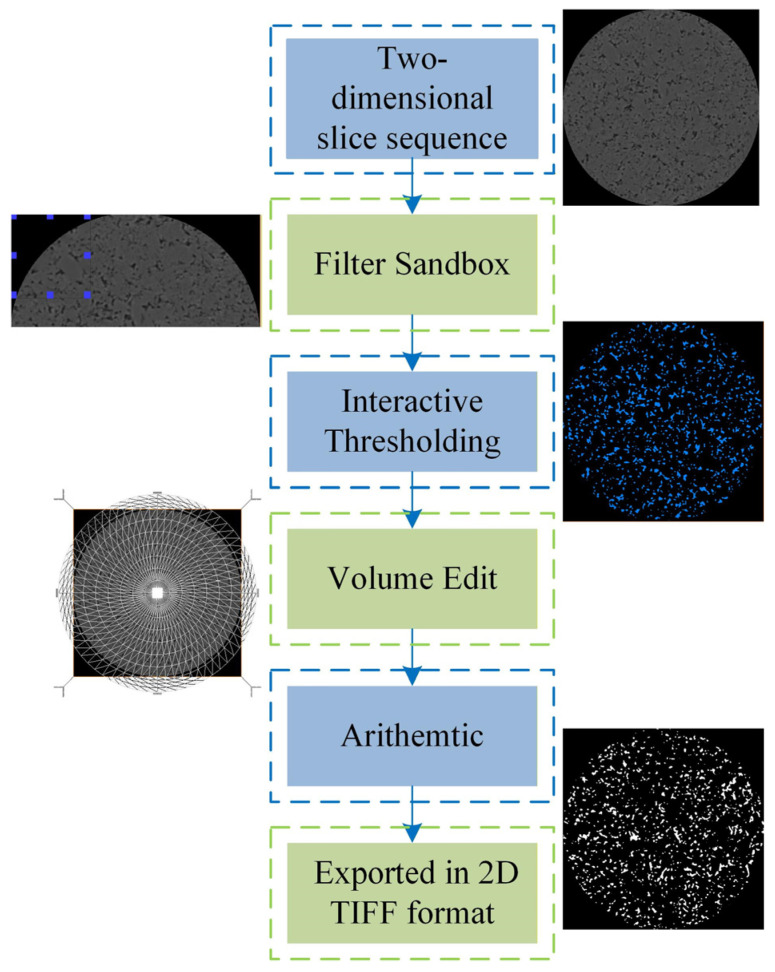
Flowchart of slice binarization.

To this end, this study implements a systematic image processing and model reconstruction workflow based on the Avizo software. The specific technical pathway for obtaining the binarized image is illustrated in Figure 2. This process encompasses all critical steps from importing the 2D slices, applying filtering and denoising, performing threshold segmentation, dividing and cropping the region of interest, to finally executing the three-dimensional reconstruction. Its purpose is to progressively transform the original, noise-containing CT images into a clear, three-dimensional pore model suitable for direct numerical computation. This establishes a reliable geometric structure foundation for uncovering the underlying mesoscopic flow mechanisms.

#### 2.2.1. Image Filtering and Denoising

Image preprocessing is the primary step to ensure model accuracy. After importing the slice sequence, the original grayscale images were first cropped to eliminate ineffective stress regions introduced by scanning field-of-view limitations or sample edge effects, ensuring that the analysis focused on the effective sample volume. Subsequently, a critical filtering and noise reduction step was performed.

CT images inevitably contain noise introduced during the acquisition process. In the micro-CT scan employed in this study, the primary manifestation of noise is the blurring of pore boundaries. This is caused by factors during X-ray imaging, such as the X-ray source focal spot size, the detector point spread function, and sample scattering, which result in the boundaries between pores and the polyimide skeleton appearing as gradual transition zones rather than ideal step edges in the image. This boundary blur exists globally at all pore-skeleton interfaces and can significantly compromise the accuracy of subsequent threshold segmentation [14]. Therefore, it is necessary to select an appropriate filtering algorithm that suppresses noise while preserving the true morphology of the pores as much as possible.

To visually demonstrate the noise characteristics and the denoising effects, this study compared and analyzed two algorithms: Non-Local Means filter and Median filter. Specifically, Non-Local Means filter exhibits excellent capability in preserving details and textures [15]; it not only smoothens uniform regions but also effectively retains edges, corners, and complex textures. This characteristic is crucial for preserving the intricate pore wall structures of porous media. In contrast, the Median filter showed minimal difference between the images before and after denoising. Although the Median filter is excellent at removing impulse noise, its smoothing effect on the more common and widespread Gaussian noise in CT images is inferior to that of Non-Local Means, potentially leading to loss of detail or residual noise.

**Figure 3:**
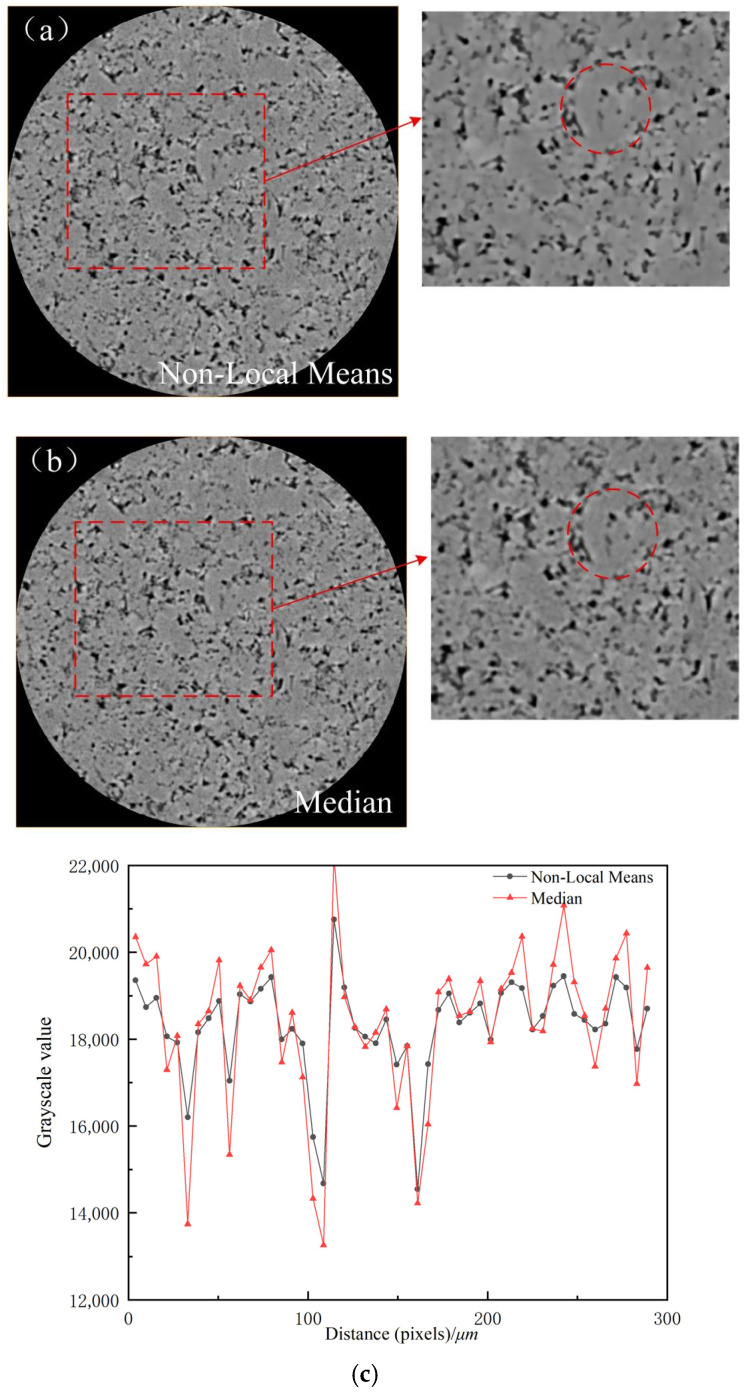

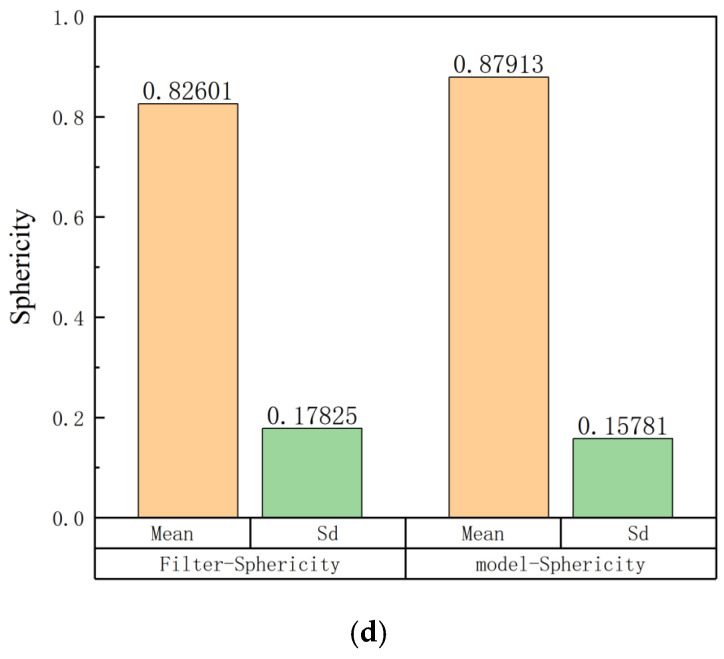
Comparison and quantitative analysis of filtering for noise reduction: (**a**) Non-Local Means; (**b**) Median; (**c**) Comparison of gray value profiles; (**d**) Comparison of mean pore sphericity and standard deviation.

Figure 3 displays the images after processing with the two filters, along with their local magnifications, and provides a visual and quantitative evaluation of the denoising effect using gray value profiles and pore sphericity analysis. Figure 3a shows the image after Non-Local Means filtering and its magnified region, where the pore boundaries appear sharp and clear, and the contrast between pores and the skeleton is significantly enhanced. Figure 3b shows the image after Median filtering and its magnified region. A comparison reveals that its boundary preservation capability is weaker, with some blurring remaining at the pore edges.

To quantitatively assess the improvement in boundary sharpness, typical pore-skeleton boundary regions at the same location in Figure 3a,b were selected. Gray value profiles were drawn perpendicular to the boundary direction, extracting the grayscale value distribution along the line. Figure 3c presents a comparison of the gray value profiles for the Non-Local Means and Median filtering methods. The results show that in the boundary region (approximately 100–200 pixels on the abscissa), the grayscale value transition after Non-Local Means filtering is steeper, and the transition zone width is significantly smaller than that after Median filtering. This indicates that Non-Local Means filtering effectively sharpens the pore boundaries, whereas the Median filter is less effective at preserving boundaries. This comparison intuitively confirms the superiority of the Non-Local Means algorithm in addressing the boundary blur noise in CT images of porous polyimide.

To verify the physical and structural accuracy of the denoised images, this study introduced pore sphericity as a quantitative metric. Sphericity is a parameter reflecting how closely the pore shape approximates a sphere; it is sensitive to boundary changes and can effectively assess whether denoising has altered the true shape of the pores. Identical threshold segmentation and pore separation (Separate Objects) processes were applied to both the original image and the image after Non-Local Means filtering. The Label Analysis module in Avizo software was then used to extract the sphericity value for each pore. To ensure statistical reliability, very small pores with a volume of less than 27 voxels (where sphericity calculation is unstable) were excluded. A total of 1523 pores were analyzed in the original image, and 1548 pores were analyzed in the filtered image.

The statistical results are shown in Figure 3d. The average sphericity of pores in the original image was 0.879 ± 0.158 (mean ± standard deviation), while that in the filtered image was 0.826 ± 0.178. The slight decrease in the mean sphericity after filtering (a relative change of approximately 6.0%) precisely demonstrates the effectiveness of denoising. In the original image, due to boundary blur, the pore boundaries were incorrectly shifted outward or inward during threshold segmentation, leading to distortion in the statistical pore morphology. This boundary blur caused an underestimation of the surface area for some pores. According to the sphericity calculation formula, an underestimated surface area leads to an overestimated sphericity value, sometimes even producing abnormal values greater than 1 (approximately 34.2% of pores in the original image exhibited sphericity > 1). After denoising, the boundaries became sharp and clear, segmentation accuracy improved, and the pore surface area was calculated correctly. Consequently, the sphericity values fell back into the true range (all pore sphericity values were less than 1). The slight decrease in the mean value reflects the correction towards the true pore morphology. The standard deviations of the two datasets are similar (0.158 vs. 0.178), and the overall distribution patterns of sphericity are essentially consistent, indicating that denoising did not alter the overall morphological characteristics of the pores but rather corrected the errors present in the original image.

Based on the comprehensive analysis above, the Non-Local Means filtering algorithm was ultimately selected for image preprocessing in this study. This algorithm can effectively suppress noise while maintaining the sharpness and morphological integrity of pore boundaries, providing a high-quality image foundation for subsequent threshold segmentation and three-dimensional reconstruction. During implementation, the search window and similarity comparison window sizes for the Non-Local Means algorithm were optimized based on image characteristics. By comparing the filtering effects (e.g., edge sharpness, changes in particle sphericity) under different parameter combinations, the final parameter set was determined. This ensured maximum preservation of pore structure details while achieving the desired denoising effect. The selected parameters are close to the standard recommended configuration in Avizo software, ensuring consistency and reliability of the processing outcome.

#### 2.2.2. Threshold Segmentation and Data Conversion

**Figure 4:**
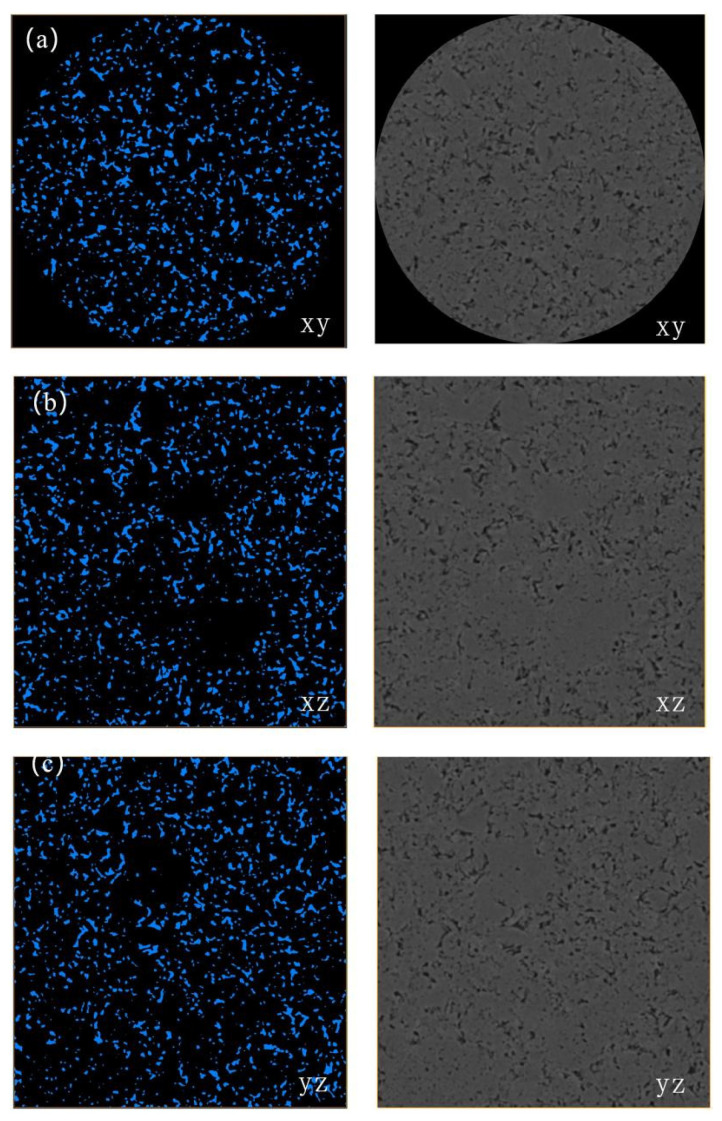
Threshold Segmentation comparison: (**a**) threshold segmentation vs. original grayscale image in the xy plane; (**b**) threshold segmentation vs. original grayscale image in the xz plane; (**c**) threshold segmentation vs. original grayscale image in the yz plane.

Following the filtering and denoising process, performing threshold segmentation on the two-dimensional CT slices is a critical step in converting continuous grayscale images into a discretized, quantitative geometric model [16]. This procedure leverages the grayscale differences between distinct material phases within the image. By applying a global or local threshold, the continuous grayscale image is transformed into a binary image containing only two phase regions: pores and the solid matrix. This achieves the conversion from imaging information to a quantitative geometric model. The accuracy of this segmentation directly determines the correctness of the geometric boundaries and the subsequent physical field calculations upon which the numerical simulations rely. As shown in Figure 4, the segmentation results displayed on orthogonal slices along the xy, xz, and yz planes demonstrate that the threshold segmentation effectively identifies and delineates the pore spaces (black) and the polyimide skeleton (white). This process generates clear binary images with well-defined boundaries, laying the structural foundation for the subsequent three-dimensional reconstruction and seepage simulation.

In the specific implementation, this study employed the Otsu adaptive threshold algorithm to automatically determine the segmentation threshold based on the global grayscale histogram. The optimal segmentation threshold for this sample was calculated to be 125 (grayscale range 0–255), a value that closely aligns with the central position of the pore-skeleton grayscale transition zone in the original image, effectively ensuring the accuracy of the segmented boundaries. Upon completion of segmentation, a morphological opening operation (using a disk-shaped structuring element with a radius of 1 pixel) was further applied to remove isolated noise points smaller than 27 voxels, thereby enhancing the cleanliness and analyzability of the binary image while preserving structural integrity.

To ensure the accuracy of the threshold segmentation results, this study conducted verification from two aspects: visual comparison and morphological rationality. First, a side-by-side comparison of the threshold-segmented image (left) and the original CT grayscale image (right), as shown for each slice in Figure 4, reveals that the segmented boundaries align closely with the pore-skeleton grayscale transition zone in the original image, without significant shift. Particularly in the magnified regions, the segmented boundaries are accurately distributed along the positions of the maximum grayscale gradient, indicating that the algorithm effectively identifies the true interface between pores and the skeleton. Second, 3D visualization based on the segmentation results (Figure 7) and morphological parameter analysis revealed that the pores predominantly exhibit irregular geometric shapes. Some pores interconnect to form networks, while others exist in isolation. The sphericity distribution is reasonable, with no anomalous structures, conforming to the typical microscopic characteristics of porous polyimide materials. This two-fold verification collectively confirms the correctness and reliability of the threshold segmentation results shown in Figure 4.

The quality of threshold segmentation has a decisive impact on subsequent LBM-based seepage simulations. On one hand, the extracted pore space can realistically reflect the actual flow paths of the fluid (lubricating oil); its morphology and connectivity directly influence the accuracy of flow field distribution, pressure gradient, and permeability prediction. On the other hand, segmentation results with clear boundaries and realistic geometry are a fundamental prerequisite for accurately calibrating wettability parameters (such as the interaction force parameter Gw). Any boundary distortion or residual noise could lead to deviations in simulating wetting behavior. Therefore, the threshold segmentation depicted in Figure 4 is not only a necessary step in the image processing workflow but also a crucial foundation for constructing reliable structure-performance correlations and deeply understanding mesoscale seepage mechanisms.

**Figure 5:**
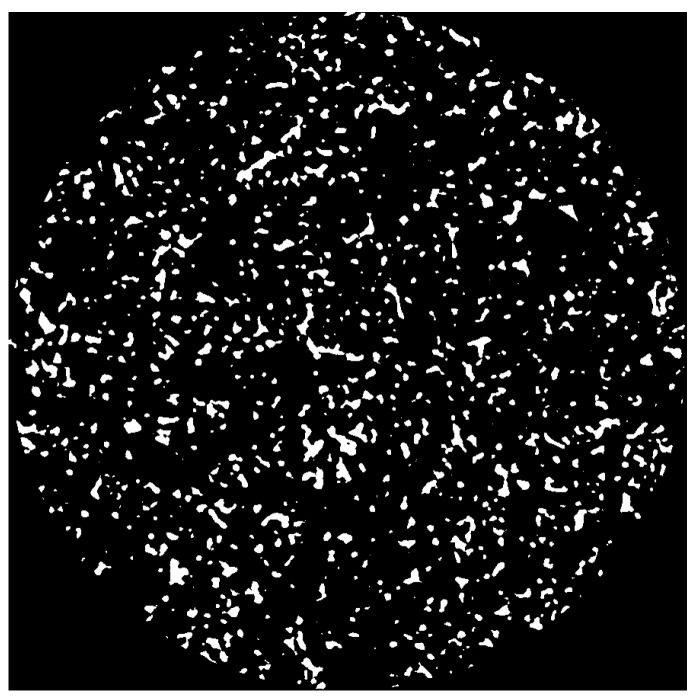
Binary images.

Following the completion of threshold segmentation, the original grayscale image (e.g., in 16-bit unsigned integer format with a range of 0–65,535) is processed, resulting in a new data module stored in memory with only two discrete values: 0 (representing pores, displayed as dark) and 1 (representing the solid matrix, displayed as light). If this data is exported and opened directly in other imaging software without further processing, pixels with a grayscale value of 1 will appear as extremely dark gray (almost black), failing to provide effective black-and-white visual contrast. This severely hampers the accurate interpretation of the pore structure. To address this issue, the Arithmetic module was employed to convert the data range of the segmentation results. Using the expression A*255, the label values in memory were transformed from {0, 1} to the standard 8-bit grayscale range {0, 255}. After this conversion, pores and the solid matrix are represented as pure black and pure white, respectively (as shown in Figure 5), providing structurally well-defined input data for subsequent 3D reconstruction and numerical analysis.

**Figure 6:**
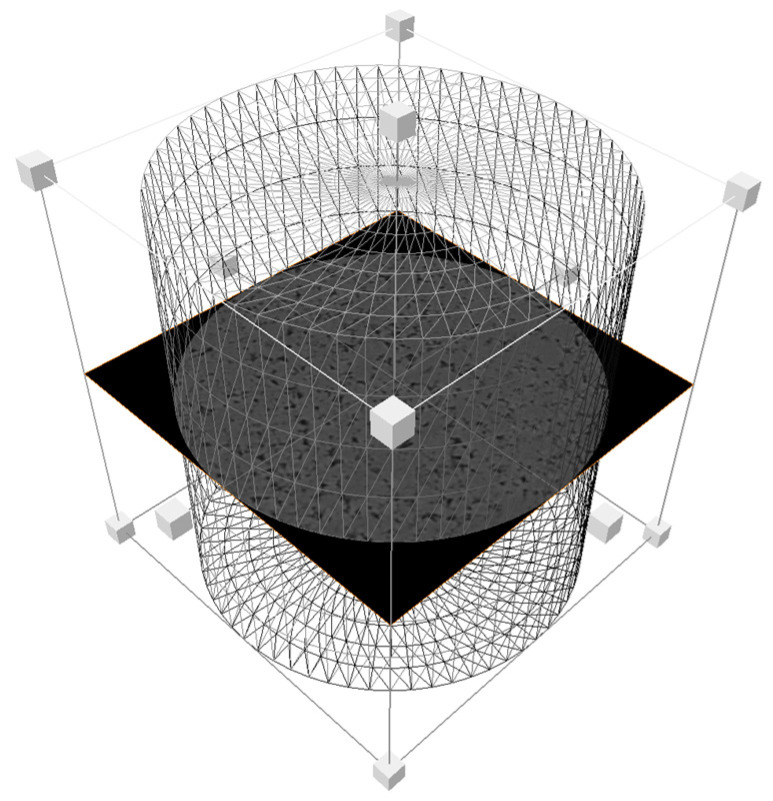
Analysis region extraction and model correction.

To further enhance the model quality, the Volume Edit module was introduced for interactive optimization of the binary image prior to exporting the final results. This module supports manually correcting erroneous voxels resulting from threshold inaccuracies and eliminating non-representative structures introduced by scanning boundaries or sample handling interference (as shown in Figure 6), thereby precisely extracting the target analysis region. This optimization step significantly improves the fidelity of the “digital core” in replicating the true geometry and topology of the porous medium, establishing a reliable structural foundation for subsequent high-fidelity seepage simulations.

### 2.3. Three-Dimensional Model Reconstruction and Visualization Analysis

Following image preprocessing and binarization, three-dimensional model reconstruction and in-depth visualization analysis were conducted based on the post-processed 2D slice sequence. This process achieved multi-modal visualization of both the original grayscale volume data and the segmented binary volume data, successfully constructing a 3D binary volumetric model capable of representing pore spaces and the solid skeleton. This provides the geometric foundation for subsequent quantitative analysis and numerical simulation.

#### 2.3.1. Construction of the 3D Voxel Model and Digital Model

**Figure 7:**
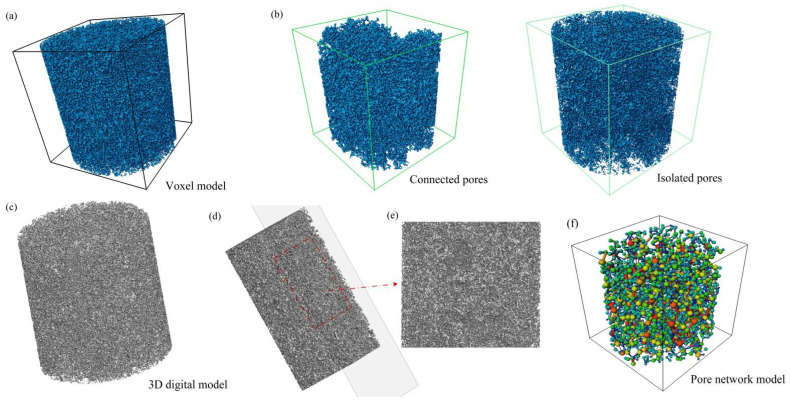
Model Construction of Porous Polyimide: (**a**) Voxel model; (**b**) Connected pores and Isolated pores; (**c**) 3D digital model; (**d**,**e**) 3D digital model cross-sectional view; (**f**) Pore network model.

The core of a three-dimensional digital model is the voxel model, which is essentially a regular three-dimensional array (grid system) where each grid point (i.e., voxel) stores one or more numerical values (e.g., grayscale value, material label) [17]. Through three-dimensional rendering techniques, an overall 3D visualization projection of the original grayscale field can be achieved (as shown in Figure 7a). However, it should be noted that this method does not generate a classical solid surface model with explicit vertex and facet structures. In contrast, the binary volume data model obtained after threshold segmentation processing achieves a digital representation of the solid concept at a discrete level by labeling each voxel as either 0 (pore) or 1 (solid skeleton).

To facilitate more flexible subsequent processing and seepage simulations, the sequence of binary images was imported into Matlab 2024 software. By stacking the 2D slice sequences layer by layer, a complete 3D matrix digital model was constructed. The volshow function was employed to perform volume rendering of this 3D data model, with the results shown in Figure 7c, intuitively reproducing the complex and interconnected three-dimensional pore structure within the porous polyimide. To gain further insight into the internal configuration of the model, Figure 7d presents a cross-sectional view of the 3D model, and Figure 7e shows a locally magnified region, clearly revealing the distribution and connectivity of pores within the porous polyimide. This completes the construction of the three-dimensional digital model of the porous polyimide structure. From the reconstruction results (Figure 7c), it can be observed that the pores exhibit irregular geometric shapes in space, with a wide pore size distribution spanning from the sub-micron to several micron scale. Pores are interconnected through narrow throats, forming an intricate network structure. This topological characteristic directly dictates the transport behavior of lubricating oil within the material.

#### 2.3.2. Quantification and Separation of Pore Structures

Three-dimensional visualization serves not only for morphological display but, more crucially, enables quantitative analysis of the microstructure. Key structural parameters, such as porosity, pore size distribution, sphericity, and pore connectivity, can be precisely obtained through calculations on the voxel model [18]. To deeply reveal the effective pathways for fluid transport, specialized analysis modules were utilized to separate and visualize the interconnected pore network from isolated pore spaces within the model, as shown in Figure 7b.

Quantitative analysis indicates significant differences in structure and function between connected and isolated pores. Connected pores are structures interconnected in three-dimensional space, forming continuous channels that serve as the primary pathways for fluid (e.g., lubricating oil) transport within the material [19]. Isolated pores, conversely, do not participate in macroscopic fluid transport. However, as important oil storage units, they play a non-negligible auxiliary role in the material’s wetting behavior and lubrication persistence. Specifically, during the initial operation or restart of a bearing, lubricant in connected pores may be partially lost due to centrifugal force or gravity. The lubricant stored in isolated pores can then act as a “strategic reservoir” or supplementary source, being slowly released via capillary action or thermal expansion to provide additional lubrication to the contact surface. Furthermore, the shape, size, and surface properties of isolated pores influence not only the initial distribution and local saturation of lubricant within the material—thereby regulating the overall lubrication efficiency—but also act as sites for microscopic stress concentration. This affects the overall strength, stiffness, and toughness of the porous polyimide skeleton, ultimately impacting the service life of the retainer under high-speed bearing loads.

#### 2.3.3. Construction of the Pore Network Model

To gain deeper insight into the key topological features governing fluid transport behavior in porous media, a corresponding pore network model (PNM) was constructed based on the reconstructed continuous pore space [20]. This was achieved using a pore network extraction technique based on the maximal ball algorithm. As shown in Figure 7c, the PNM provides a topological abstraction of the complex pore system in the form of a ball-and-stick model. This model is a simplified mathematical representation of the complex continuous pore space, where “balls” represent pore bodies and “sticks” represent connecting throats. This abstraction effectively strips away the intricate geometric details of the original pore space, instead extracting and preserving the key topological parameters (such as pore connectivity, coordination number, topological dimension) and geometric parameters (such as throat radius, pore body volume, shape factor) that dominate fluid seepage behavior.

It should be noted that the pore network model (Figure 7f) is presented alongside the aforementioned geometric models (Figure 7a–e) in Figure 7 to comprehensively illustrate the complete technical workflow, from raw data to geometric reconstruction and finally to topological abstraction. The original grayscale volume data in Figure 7a underwent threshold segmentation to yield the binary volume data model (Figure 7c), from which connected and isolated pores were separated (Figure 7b). Cross-sectional views (Figure 7d,e) further reveal the internal pore structure. Building upon this foundation, the pore network model (Figure 7f) extracts the key topological parameters governing seepage behavior, achieving a transition from geometric structure to seepage function.

The construction of the pore network model provides a powerful theoretical tool for bridging the gap from geometric structure to seepage function. Its simplified structure significantly reduces the computational complexity of pore-scale multiphase flow simulations, making systematic studies of key seepage issues—such as capillary pressure curves, relative permeability prediction, and immiscible displacement dynamics—feasible. Moreover, this model can intuitively depict preferential flow paths and residual phase distribution within the pore space, contributing to a deeper understanding of mesoscopic seepage mechanisms like wettability and interfacial effects [21]. Therefore, as a crucial bridge connecting microscopic pore structure to macroscopic transport properties, the pore network model lays a solid foundation for fundamentally revealing and optimizing the lubrication and transport performance of porous polyimide materials.

**Figure 8:**
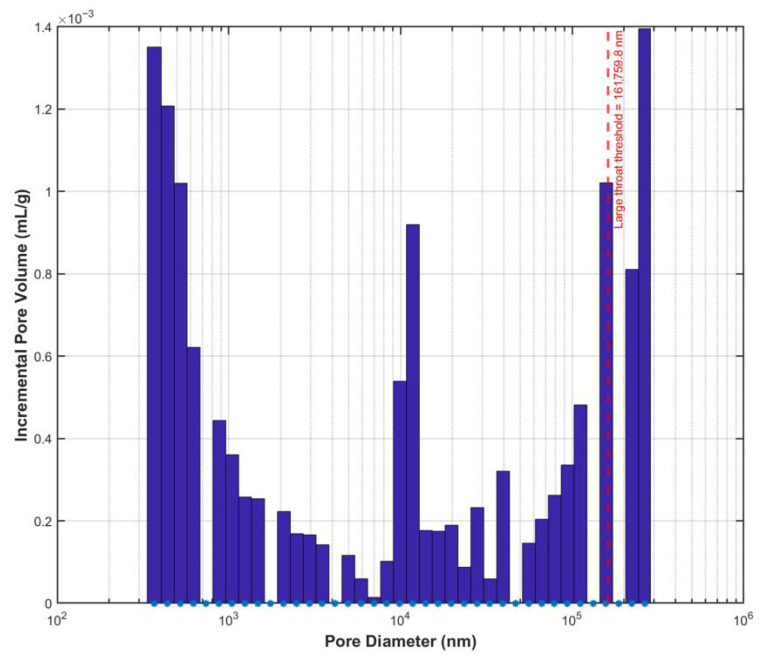
Histogram of pore-throat size distribution.

To quantitatively characterize the throat size distribution consistent with the CT model scale, mercury intrusion porosimetry (MIP) was employed to test the sample, and the intrusion pressure was converted to throat diameter using the Washburn equation. Considering the CT resolution of 300 nm, only throat data with diameters ≥ 300 nm were retained, and their volume distribution histogram was plotted (Figure 8). Based on the volume-weighted mean pore diameter (μ) and standard deviation (σ), “large throats” were defined as those with diameters greater than μ+σ, corresponding to a threshold value of 5383.2 nm (indicated by the red dashed line in Figure 8). Statistical analysis reveals that although these large throats account for only approximately 12% of the total number of CT-resolvable throats, they contribute over 65% of the total pore volume. This intuitively confirms the structural characteristic where “a few large throats dominate the pore volume,” providing a quantitative basis for subsequent analysis of preferential seepage pathways.

Based on the pore network model and the MIP experimental data, the extracted key structural parameters are listed in Table 2. Building upon this, the adequacy of the 300 nm scanning resolution selected in this study for pore structure characterization can be further verified. Figure 8 shows that the primary throat diameters are all greater than 300 nm, with an average throat diameter of 624.7 nm and a large throat threshold reaching 5383.2 nm. This indicates that the key transport channels are composed of at least 2 × 2 × 2 voxels, enabling stable identification and effectively avoiding structural distortion caused by partial volume effects. Furthermore, the pore sphericity analysis in Section 2.2.1 demonstrates clear pore boundaries and accurate segmentation results after image preprocessing. Concurrently, the scanned volume significantly exceeds the representative elementary volume of the porous medium, ensuring the reliability of the statistical results. In summary, the 300 nm resolution fully satisfies the requirements for pore structure characterization in this study.

## 3. Lattice Boltzmann Model Construction and Validation

### 3.1. Theoretical Framework of the Lattice Boltzmann Method

The Lattice Boltzmann Method (LBM) is a computational fluid dynamics technique based on mesoscopic kinetic theory. Its core concept lies in the statistical description of the microscopic motion of fluid particle ensembles. By discretizing the velocity, space, and time dimensions and restricting computational particles to specific grid nodes, it performs macroscopic statistics on particles with specific probabilities [11]. Precisely due to this mesoscopic scale characteristic, LBM avoids the difficulty of macroscopic mathematical-physical models in inadequately describing microscopic motion, while overcoming the drawbacks of molecular dynamics simulations, such as enormous computational cost and difficulty in scaling to microscopic domains. Consequently, it has become an effective tool for simulating complex flows within porous media.

This section will systematically introduce the fundamental theoretical framework of LBM, including the Boltzmann equation with the BGK approximation, the selection of the discrete velocity model (D3Q27), the introduction of the multiple-relaxation-time (MRT) model, and the moment-space transformation method. This lays the theoretical foundation for subsequent seepage simulations based on the realistic pore structure.

#### 3.1.1. Boltzmann Equation and BGK Approximation

The theoretical foundation of LBM originates from the Boltzmann equation in statistical mechanics, which accurately describes the evolution of the distribution of gas molecules in phase space. From a methodological perspective, LBM is a model derived by combining the Boltzmann equation with a discrete lattice model on the basis of lattice gas automata (LGA), offering both rigor and computational efficiency [22]. For a single-component gas, the velocity distribution function is denoted as f, which is a function of the spatial position vector r(x,y,z), the molecular velocity vector $\xi (\xi_{x},\xi_{y},\xi_{z})$, and time t. f(r,ξ,t)drdξ represents the number of molecules at time t within the volume element dr=dxdydz between r and r+dr, with velocities between ξ and ξ+dξ. The complete form of the Boltzmann equation is as follows [23]:

(1) $$\frac{\partial f}{\partial t}+\xi \cdot \frac{\partial f}{\partial r}+a\cdot \frac{\partial f}{\partial \xi }=\iint \left(f^{\prime }f_{1}^{\prime }-ff_{1}\right)d_{D}^{2}|g|\cos\theta d\Omega d\xi_{1}$$

(2) $$\Omega_{f}=\iint \left(f^{\prime }f_{1}^{\prime }-ff_{1}\right)d_{D}^{2}|g|\cos\theta d\Omega d\xi_{1}$$

where a is the acceleration due to external forces; $d_{D}$ is the diameter of the two molecules; g is the relative velocity of the molecules; θ is the collision angle between the two molecules; dΩ is the solid angle element on the sphere; and $\Omega_{f}$ is the collision term, describing the change in the distribution function caused by intermolecular collisions.

The Boltzmann equation is a complex integro-differential equation. Its collision term $\Omega_{f}$ on the right-hand side involves an intricate integral form, making direct solution extremely difficult. To address this, Bhatnagar, Gross, and Krook proposed the BGK (Bhatnagar–Gross–Krook) approximation model [24], which linearizes the collision term into a single relaxation form:

(3) $$\Omega_{f}=v\left[f^{eq}\left(r,\xi \right)-f\left(r,\xi ,t\right)\right]$$

where kinematic viscosity $v=\frac{1}{\tau }$, with τ being the relaxation time, controlling the rate at which the distribution function approaches equilibrium; and $f^{eq}$ is the local equilibrium distribution function, usually taken as the Maxwell–Boltzmann distribution. This leads to the Boltzmann-BGK equation:

(4) $$\frac{\partial f}{\partial t}+\xi •\frac{\partial f}{\partial r}+a•\frac{\partial f}{\partial \xi }=v(f^{eq}-f)$$

However, although the BGK model simplifies computation and has greatly promoted the practical application of LBM, it is prone to numerical instability in flows with high Reynolds numbers or significant non-equilibrium characteristics. Therefore, the multiple-relaxation-time (MRT) model was subsequently developed to enhance stability.

#### 3.1.2. Discrete Velocity Model and the D3Q27 Scheme

**Figure 9:**
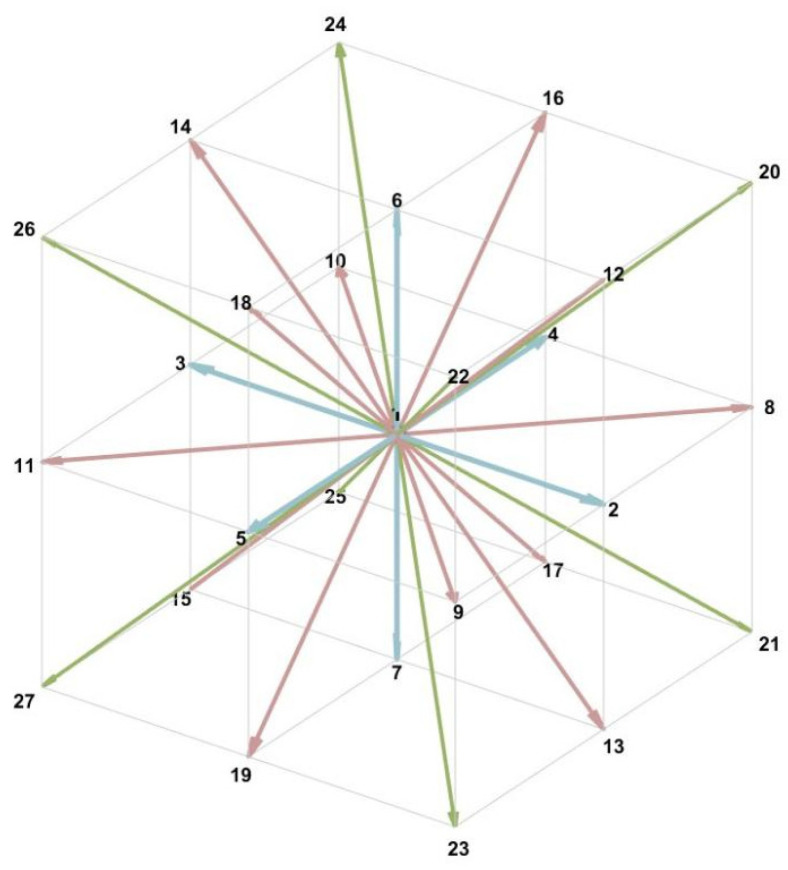
D3Q27 discrete velocity model.

To further achieve numerical solution, it is necessary to discretize the velocity space, physical space, and time. The DdQm (d-dimensional space with m discrete velocities) series of models, proposed by Qian et al. as early as 1992 [25,26,27], serves as the fundamental framework for the Lattice Boltzmann method. Common three-dimensional discrete velocity models include D3Q15, D3Q19, and D3Q27. This study employs the D3Q27 discrete velocity model (as shown in Figure 9) due to its more comprehensive set of velocity directions. This enables a more accurate description of non-equilibrium behavior within complex pore geometries, significantly reduces grid orientation errors, and enhances numerical stability under high Reynolds number conditions [24]. Although computationally more expensive, its advantages in handling curved pore walls and inclined flow paths within porous media are significant, aligning with this study’s dual requirements for accuracy and stability. The model defines 27 discrete velocity directions $e_{i}$ (see Table 3) in three-dimensional space and introduces corresponding weight coefficients $w_{i}$, thereby establishing the discrete velocity set $\left\{e_{i}\right.,{\left.w_{i}\right\}}_{i=0}^{26}$.

#### 3.1.3. Multiple-Relaxation-Time Model (MRT-LBM)

Although the BGK model is structurally simple and easy to implement, allowing it to achieve essentially second-order numerical accuracy by simply specifying a different equilibrium distribution function and directly adding external force terms, it employs only a single relaxation time, making it difficult to simultaneously ensure both numerical stability and physical accuracy. Furthermore, Lallemand et al. [28] demonstrated that (when the relaxation frequency is greater than 1.92; i.e., the relaxation time approaches 0.5), particularly in flows with high Reynolds numbers or pronounced non-equilibrium states, divergence issues easily occur. To address this, d’Humières et al. [29] proposed the multiple-relaxation-time (MRT) model (the introduction of the MRT collision operator was almost concurrent with that of the BGK collision operator). This model allows different distribution functions to have different relaxation times in moment space, thereby mitigating the numerical instabilities common in high-Reynolds-number flows and significantly improving the model’s numerical stability and physical applicability.

The evolution equation of the MRT model consists of two steps: collision and streaming. In the collision step, the distribution functions relax in moment space:

(5) $$m^{*}\left(x,t\right)=m\left(x,t\right)-S•\left[m\left(x,t\right)-m^{eq}\left(x,t\right)\right]$$

where m=M•f is the moment vector, mapping the distribution function from velocity space to moment space; S is the diagonal relaxation matrix, with each relaxation parameter $s_{i}$ controlling the relaxation rate of density, momentum, energy, and higher-order moments, respectively; $m^{eq}$ is the equilibrium moment vector, determined by the macroscopic density ρ and velocity u:

(6) $$m^{eq}=m^{eq}\left(\rho ,u\right)$$

The streaming step remains consistent with the BGK model and takes the form:

(7) $$f_{i}\left(x+e_{i}\delta t,t+\delta t\right)=f_{i}^{*}\left(x,t\right)$$

By combining the collision and streaming processes, the complete D3Q27-MRT evolution equation is obtained:

(8) $$f_{i}\left(x+e_{i}\delta t,t+\delta t\right)=f_{i}\left(x,t\right)-M^{-1}•S•\left[m\left(x,t\right)-m^{eq}\left(x,t\right)\right]$$

In the D3Q27 model, the equilibrium distribution function adopts the following form:

(9) $$f_{i}^{eq}\left(x,t\right)=\omega_{i}\rho \left(x,t\right)\left[1+\frac{e_{i}•u^{eq}}{c_{s}^{2}}+\frac{{\left(e_{i}•u^{eq}\right)}^{2}}{2c_{s}^{4}}-\frac{{\left(u^{eq}\right)}^{2}}{c_{s}^{2}}\right]$$

where $u^{eq}$ is the fluid velocity vector on the lattice scale; $c_{s}=\frac{1}{\sqrt{3}}$ is the lattice speed of sound. The macroscopic density and velocity can be recovered from the moments of the distribution functions:

(10) $$\rho =\sum_{i=0}^{26}f_{i},\rho u=\sum_{i=0}^{26}f_{i}e_{i}$$

Furthermore, the kinematic viscosity ν of the fluid and the relaxation parameters satisfy the following relation:

(11) $$v=c_{s}^{2}\left(\frac{1}{s_{v}}-\frac{1}{2}\right)\delta t$$

where $s_{v}$ is the relaxation parameter in the relaxation matrix S that controls the viscous stress.

#### 3.1.4. Moment Transformation and Relaxation Matrix

The core of the MRT model lies in the moment space transformation, achieved through the transformation matrix M and the diagonal relaxation matrix S.

1. Transformation Matrix M:

The transformation matrix M is a 27 × 27 matrix. Its function is to linearly transform the distribution function vector f from discrete velocity space to moment space m:

(12) m=M•f

Each component of the moment vector mm corresponds to a macroscopic physical quantity of the fluid or its higher-order moments. For the D3Q27 model, these moments typically include conserved moments (such as density ρ, momentum $j_{x},j_{y},j_{z}$) and non-conserved moments (such as energy e, energy square ε, energy flux $q_{x},q_{y},q_{z}$, stress tensor components $p_{xx},p_{ww},p_{xy},p_{y2},p_{x2}$ and higher-order moments). Constructing the transformation matrix MM through the Gram-Schmidt orthogonalization process ensures the physical orthogonality and independence of these moments. This forms the basis for the MRT model’s ability to independently adjust the relaxation rates of different modes.

2. Relaxation Matrix S:

The relaxation matrix S is a 27 × 27 diagonal matrix:

(13) $$S=diag\left(s_{0},s_{1},s_{2},\ldots ,s_{26}\right)$$

The elements $s_{\alpha }$ on its diagonal are the relaxation rates, which respectively control the relaxation process of the corresponding components in the moment vector m. The relaxation rates corresponding to conserved quantities (density, momentum) are typically set to 0, meaning these moments remain unchanged during collision. The relaxation rate $s_{v}$ associated with viscous stress directly determines the fluid’s kinematic viscosity, as shown in Equation (11). The relaxation rates for the remaining higher-order moments are treated as free parameters. By adjusting these relaxation rates to their optimal values (usually close to 1), the numerical stability of the model can be maximized without affecting the macroscopic Navier–Stokes behavior.

3. Inverse Transformation:

After the collision step is performed in moment space, the updated moment vector $m^{*}$ needs to be transformed back to distribution function space via the inverse matrix $M^{-1}$ of M to proceed with the subsequent streaming step:

(14) $$f^{*}=M^{-1}\cdot m^{*}$$

The configuration of the moment transformation and relaxation matrix is key to the MRT model’s ability to independently control the relaxation behavior of each order of moments. Through reasonable setting of the relaxation parameters, numerical stability can be significantly enhanced while ensuring macroscopic Navier–Stokes behavior. The D3Q27-MRT model adopted in this study, by optimizing the diagonal elements of the relaxation matrix, ensures convergence and accuracy in seepage simulations within the complex pore structure of porous polyimide. This provides a reliable numerical tool for subsequent wettability parameter calibration and dynamic process simulation.

### 3.2. Boundary Condition Setting and Model Validation

To verify the correctness and reliability of the D3Q27 multiple-relaxation-time (MRT) lattice Boltzmann model constructed in Section 3.1, a systematic numerical validation is required. The core of the validation scheme is to select classical flow benchmark cases with analytical solutions or high-precision reference solutions, in order to test the model’s accuracy, stability, and boundary treatment capability when simulating different types of flows. This section first elaborates on the methods for setting boundary conditions, followed by validation using two benchmark cases: lid-driven cavity flow and Poiseuille flow.

#### 3.2.1. Boundary Condition Setting

In the lattice Boltzmann method, the accurate setting of boundary conditions is crucial for the success of numerical simulations, directly affecting the physical authenticity of fluid-solid interaction and computational stability. Boundary conditions in this method are typically imposed in the form of macroscopic quantities (such as pressure, velocity, temperature, etc.). The boundary schemes can be mainly categorized into heuristic schemes, kinetic schemes, extrapolation schemes, and other complex boundary treatment formats [24]. Based on boundary type, LBM boundary conditions can be classified into velocity boundaries (including flat and curved boundaries) and pressure boundaries, in addition to specially defined boundaries such as inlet boundaries, outlet far-field boundaries, and symmetric boundaries, among others. Properly designing and implementing boundary conditions is a key and indispensable step in the LBM simulation process. For the complex porous medium structure studied in this paper, and to ensure computational efficiency while accurately handling its intricate solid wall boundaries, this study adopts two validated boundary schemes: the standard bounce-back scheme and the non-equilibrium extrapolation scheme.

1. Standard Bounce-Back Scheme:

In the Lattice Boltzmann Method (LBM), one of the most common approaches for handling stationary solid boundaries is the standard bounce-back scheme. This method simulates the no-slip solid wall condition by directly reflecting particles that collide with the boundary back in the opposite direction. It is one of the most classical and numerically robust boundary treatment schemes in LBM. Its physical concept is intuitive, and it offers high computational efficiency, making it suitable for flow problems involving complex geometric structures.

**Figure 10:**
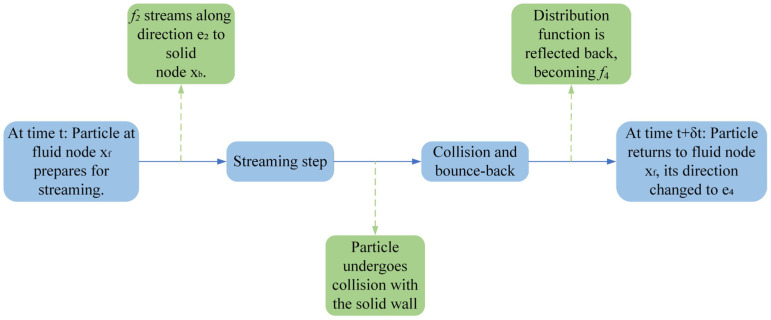
Schematic diagram of the standard bounce-back process.

Although the standard bounce-back scheme is theoretically only first-order accurate [30], its advantages lie in its excellent numerical stability, strong robustness, relatively low computational cost, and strict adherence to the laws of mass and momentum conservation. These characteristics make it particularly important for handling porous media with complex topological structures. Furthermore, studies have shown that when simulating complex porous media, the standard bounce-back scheme often outperforms many higher-order schemes in terms of computational efficiency and conservation properties [31]. The microscopic mechanism of this method can be briefly described as follows: when a fluid particle moves along a discrete velocity direction and reaches a solid node, it is considered to undergo an instantaneous collision with the wall and is then reflected back into the fluid region in the exact opposite direction. This process is illustrated in Figure 10. The entire sequence is completed within a single time step, encompassing three consecutive stages: “streaming–collision–bounce-back.”

The mathematical description of this process is as follows. At the fluid node $x_{f}$, the bounce-back behavior can be uniformly expressed as:

(15) $$f_{i}\left(x_{f},t+\Delta t\right)=f_{i}\left(x_{f},t\right)$$

where $e_{i}^{-}=-e_{i}$. Taking the scenario depicted in Figure 9 as an example, the corresponding specific expression is:

(16) $$f_{4}\left(x_{f},t+\Delta t\right)=f_{2}\left(x_{f},t\right)$$

It is worth noting that the multiple-relaxation-time (MRT) model employed in this work inherently possesses favorable numerical stability characteristics. The simplicity of the standard bounce-back scheme, combined with the tunability of the MRT model, together construct a highly stable and reliable numerical computational framework. This effectively supports the precise simulation of long-term transient seepage processes within high-resolution, large-scale porous media.

2. Non-Equilibrium Extrapolation Scheme:

Inspired by Chen’s extrapolation scheme and Zou’s non-equilibrium bounce-back scheme, Guo et al. proposed a new boundary treatment format in 2002, namely the non-equilibrium extrapolation scheme [32]. Its fundamental concept is to decompose the distribution function on the boundary node into equilibrium and non-equilibrium components. The equilibrium part is approximated based on the definition of the boundary conditions, while the non-equilibrium part is determined via non-equilibrium extrapolation. This scheme offers second-order numerical accuracy. It can precisely position the actual boundary location at the solid node itself, thereby enabling a more accurate representation of curved pore-wall geometries. This capability is crucial for analyzing the micro-effects of surface roughness on the flow.

**Figure 11:**
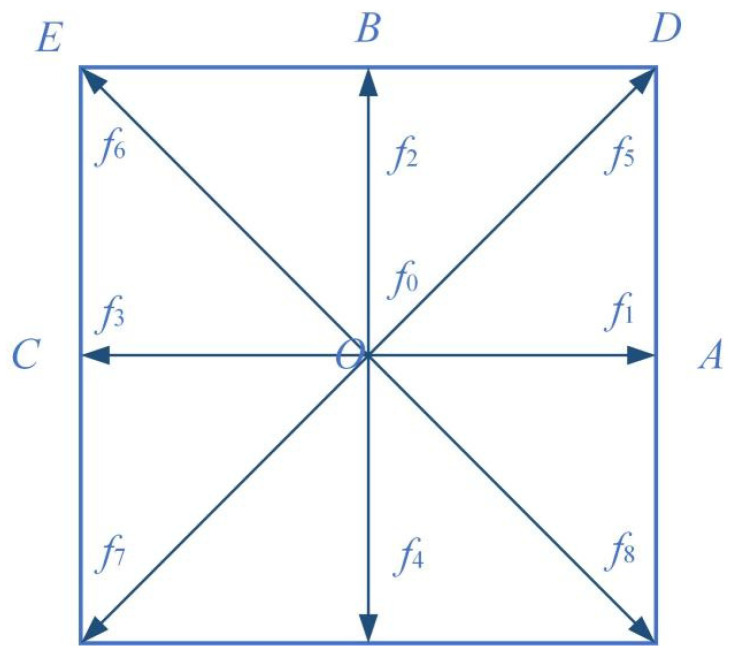
Non-equilibrium extrapolation scheme.

The core mechanism of this scheme lies in physically decomposing the distribution functions at boundary nodes based on kinetic theory, and drawing on the theoretical ideas of the Chapman–Enskog expansion to apply correspondingly reasonable approximations to different components, thereby achieving high-precision implementation of boundary conditions. As shown in Figure 11, O represents a boundary node located at the computational domain boundary (i.e., point COA), B represents an adjacent fluid node (i.e., point EBD), and A, C, D, E are other adjacent fluid nodes; *f*_0_ to *f*_8_ denote the distribution functions in the discrete velocity directions, where solid arrows represent known distribution functions (which can be provided by fluid nodes), and dashed arrows represent the distribution functions to be determined (which need to be obtained via interpolation) [33]. Before the collision step, the time-dependent distribution functions at the boundary node O need to be obtained through an interpolation scheme, with the specific expression given as:

(17) $$f_{a}\left(O,t\right)=f_{a}^{eq}\left(O,t\right)+f_{a}^{neq}\left(O,t\right)$$

For the equilibrium distribution $f_{a}^{eq}\left(O,t\right)$, it can be obtained using the macroscopic physical quantities at the boundary node, i.e., approximating the density at the wall with the density of the adjacent fluid node in the equilibrium function:

(18) $$f_{a}^{eq}\left(O,t\right)\approx F^{eq}\left[\rho \left(B,t\right),u\left(O,t\right)\right]$$

For the non-equilibrium part $f_{a}^{neq}\left(O,t\right)$, the non-equilibrium distribution function at point *B* can be used for approximation:

(19) $$f_{a}^{neq}\left(B,t\right)=f_{a}\left(B,t\right)-F^{eq}\left[\rho \left(B,t\right),u\left(B,t\right)\right]$$

Meanwhile, considering the relationship between the non-equilibrium distribution functions at the boundary node *O* and the adjacent interior node *B*:

(20) $$f_{a}^{neq}\left(B,t\right)=f_{a}^{neq}\left(O,t\right)+O\left(\delta_{a}^{2}\right)$$

where $\delta_{a}$ is the spatial step size. Therefore, the non-equilibrium part at point *B* can be used to approximate the non-equilibrium part at point *O*. Based on the theoretical derivation above, the distribution function at the boundary node *O* can be approximated by the following equation:

(21) $$f_{a}\left(O,t\right)=f_{a}^{eq}\left(O,t\right)+\left[f_{a}\left(B,t\right)-f_{a}^{eq}\left(B,t\right)\right]$$

If the collision process is considered, the post-collision distribution function at point *O* can be expressed as:

(22) $$f_{a}^{+}\left(O,t\right)=f_{a}^{eq}\left(O,t\right)+\left(1-\frac{1}{\tau }\right)\left[f_{a}\left(B,t\right)-f_{a}^{eq}\left(B,t\right)\right]$$

#### 3.2.2. Lid-Driven Cavity Flow Validation

**Figure 12:**
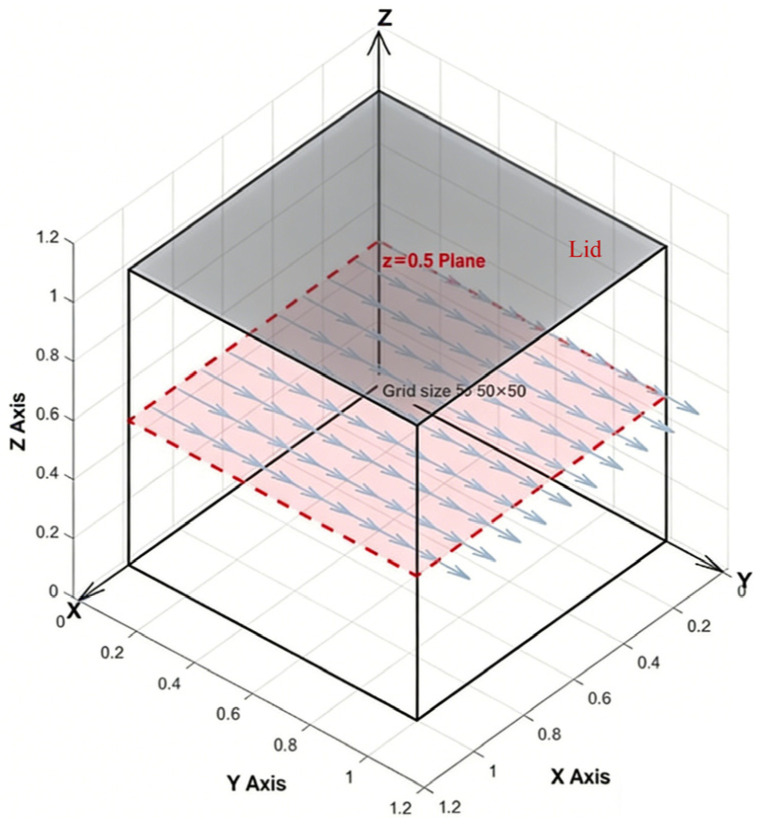
Schematic diagram of the three-dimensional lid-driven cavity flow.

Lid-driven cavity flow is a classic benchmark case widely used in computational fluid dynamics to evaluate the accuracy, stability, and boundary handling capability of numerical methods for incompressible flows [24]. The physical model is illustrated in Figure 12, where a constant tangential velocity (along the positive y-direction) is imposed on the top wall (i.e., the driving lid), while the remaining walls are kept stationary. Driven by the shear of the lid, a complex vortical structure comprising primary and secondary vortices develops within the cavity. These flow characteristics provide a clear assessment of the precision and robustness of numerical schemes.

To verify the correctness of the D3Q27-MRT model constructed in this study, a three-dimensional flow simulation was conducted in a cubic computational domain with dimensions of 50 × 50 × 50, and the results were compared with literature data. The boundary conditions were set as follows: the lid moves in the positive y-direction, its velocity adjusted according to the target Reynolds number; the other five walls are stationary no-slip solid walls. The initial density of the flow field was set to $\rho_{0}=1.0$ and the initial velocity was zero. By varying the lid velocity, multiple conditions with Reynolds numbers Re=UL/ν of 100, 200, 400, 1000, and 2000 were realized on the central cross-section z=Lz/2, in order to examine the model’s performance in simulating flows from laminar to transitional regimes. All solid wall boundaries were treated using the standard bounce-back scheme described in Section 3.2.1.

**Figure 13:**
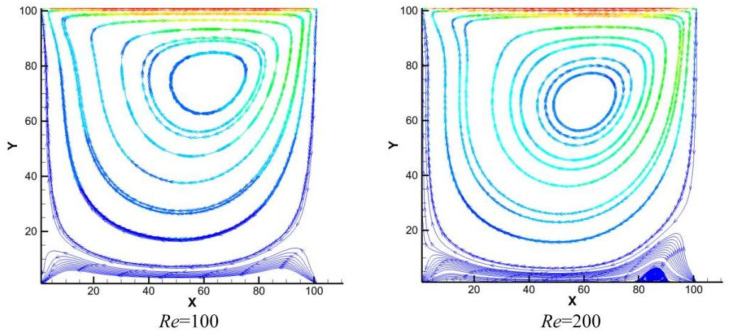
Streamline distribution diagrams for Re = 100 and 200. (The colors represent velocity magnitude, with red indicating high-velocity regions and blue indicating low-velocity regions; flow direction is indicated by arrows).

Figure 13 shows the streamline distribution characteristics on the z/2 cross-section at Reynolds numbers of 100 and 200. Combined with quantitative analysis, the vortex differences under different conditions can be more clearly illustrated. As seen in the figure, at Re = 100, the streamlines rotate around a single center, forming a dominant central vortex without significant flow separation or additional vortices. From a quantitative perspective, the primary vortex occupies over 85% of the flow area on the cross-section. The curvature of the streamlines is uniform, with fluctuation amplitudes less than 5%, and no local streamline reversal is observed. This is because the flow is in a laminar state at this point, with weaker shear forces (approximately 0.4 times that at Re = 200), making it difficult to generate significant secondary vortices. This aligns with classical research conclusions; for instance, studies by Ghia et al. [34] indicate that when Re ≤ 100, only the primary vortex exists in lid-driven cavity flow, and secondary vortices have not yet developed. When the Reynolds number increases to 200, the streamlines at the lower-left and lower-right corners of the primary vortex show slight curvature and signs of separation. The local curvature fluctuation amplitude of the streamlines increases to 15–20%, but the coverage area of the secondary vortices is less than 10% of the cross-section, and their structure is not yet stable. At this stage, the flow gradually transitions from laminar to transitional. The shear force is approximately 1.5 times greater than at Re = 100, potentially inducing weak secondary vortices in the corner regions. However, this structure is far less pronounced than at Re ≥ 400 (where secondary vortex coverage can exceed 25%). Based on the features in the figure, it can be concluded that under this condition, the secondary vortices are not fully formed and remain in a nascent state.

**Figure 14:**
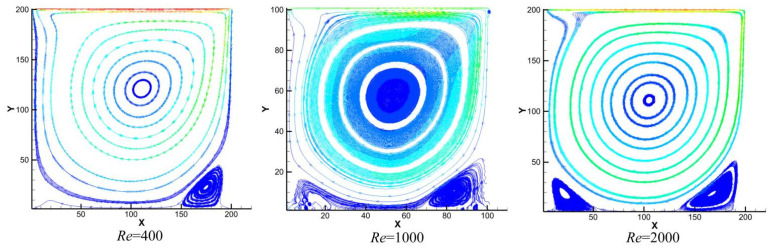
Streamline distribution diagrams for Re = 400, 1000, and 2000. (The colors represent velocity magnitude, with red indicating high-velocity regions and blue indicating low-velocity regions; flow direction is indicated by arrows).

Figure 14 displays the streamline distributions on the z/2 cross-section at Reynolds numbers of 400, 1000, and 2000, clearly revealing the evolution process of the vortex structure. At Re = 400, a distinct, closed independent secondary vortex first forms in the lower-right corner of the cavity, marking the transition of the flow from simple laminar to a complex multi-vortex structure. This is consistent with the conclusions of classical studies such as Ghia et al. As the Reynolds number increases to 1000, the flow enters a turbulent transition stage. The inertial effects and shear forces are significantly enhanced, giving rise to the embryonic form of a smaller-scale, compact tertiary vortex in the lower-left corner. This exemplifies the cascade process where energy transfers from larger to smaller scales. By Re = 2000, the multi-level vortex structure becomes more stable and well-developed. Stable, multi-scale vortex clusters, containing both secondary and tertiary vortices, form in both the bottom left and right corners. The flow field exhibits characteristics typical of a fully developed transitional state.

In summary, based on the flow field structure presented in Figure 14, this study clearly captures the classic evolutionary path of lid-driven cavity flow with increasing Reynolds number: “stable development of secondary vortices → asymmetric emergence of tertiary vortices → final formation of a multi-scale vortex structure.” This process is jointly governed by inertial effects and the energy cascade mechanism. The results show high agreement with authoritative benchmark solutions, which not only verifies the model’s accuracy but also completely depicts the physical progression of the flow from a laminar-transitional state to a complex multi-scale structure.

It should be noted that this study conducted three-dimensional lid-driven flow simulations (50 × 50 × 50 grid), but the streamline distribution at the central plane (z = Lz/2) was selected for qualitative comparison with the classical two-dimensional benchmark solution (Ghia et al. [34]). The rationale for this approach lies in the fact that, within the moderate Reynolds number range, the flow structure at the central plane of a three-dimensional cavity exhibits a high degree of qualitative similarity to two-dimensional cavity flow, particularly in terms of the morphology and evolution trends of the primary and secondary vortices. Therefore, comparison with the two-dimensional results effectively validates the model’s capability to capture complex boundary-driven flows. The quantitative accuracy of the present model has been verified through the Poiseuille flow benchmark (which possesses an exact analytical solution), as detailed in Section 3.2.3. Together, these two validations constitute a comprehensive model verification framework.

#### 3.2.3. Poiseuille Flow Validation

As a classical problem in fluid mechanics, Poiseuille flow possesses an exact analytical solution and is frequently employed to validate the accuracy of numerical methods [35]. Based on the D3Q27 multiple-relaxation-time (MRT) lattice Boltzmann model constructed in Section 3.1, this study simulates Poiseuille flow within a three-dimensional cylindrical channel to systematically verify the model’s reliability for pressure-driven flows. Furthermore, it analyzes the incompressible characteristics and density stability of the lubricating oil under low Mach number conditions.

**Figure 15:**
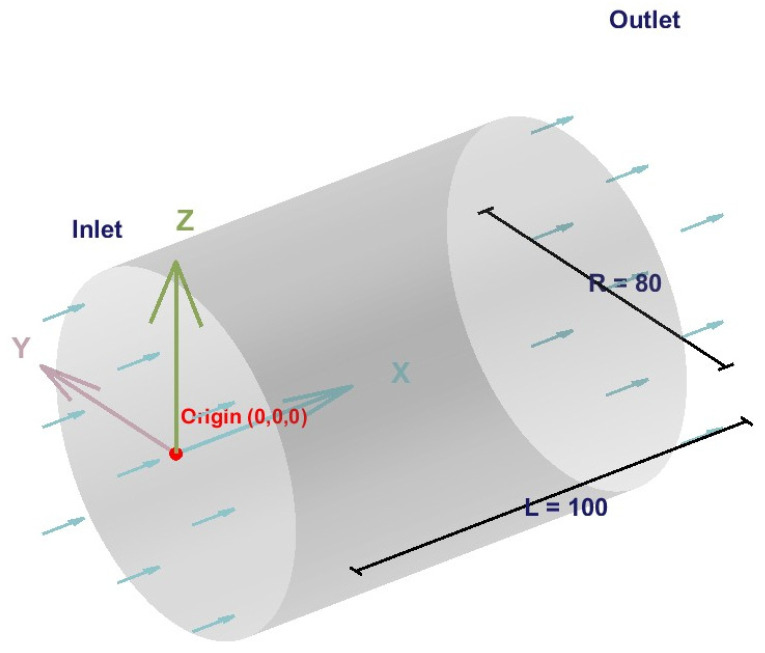
Schematic diagram of three-dimensional cylindrical Poiseuille flow.

A three-dimensional cylindrical channel model, as illustrated in Figure 15, was constructed with grid dimensions Lx = 100, Ly = Lz = 40. The Zou–He pressure boundary condition [10] was applied by specifying densities at the inlet and outlet, strictly controlling the density difference within 0.1%. The resulting pressure gradient of magnitude $\Delta p=3.33\times 10^{-4}$ served as the driving mechanism for the flow. A Reynolds number of Re = 100 was set to simulate the flow state under practical operating conditions. The fluid kinematic viscosity was achieved by adjusting the relaxation time to ensure the stability of the numerical simulation.

The analytical solution formula for three-dimensional cylindrical Poiseuille flow is:

(23) $$u\left(r\right)=\frac{\Delta p}{4\mu L}\left(R^{2}-r^{2}\right)$$

where Δp is the pressure difference driving the fluid; μ is the dynamic viscosity of the fluid; L is the length of the cylinder; R is the pipe radius, a constant; and r is the radial coordinate, a variable that determines the velocity variation at a specific location on the cross-section. From the analytical solution formula, it is evident that the velocity distribution of Poiseuille flow along the flow direction follows a parabolic profile across the cross-section.

**Figure 16:**
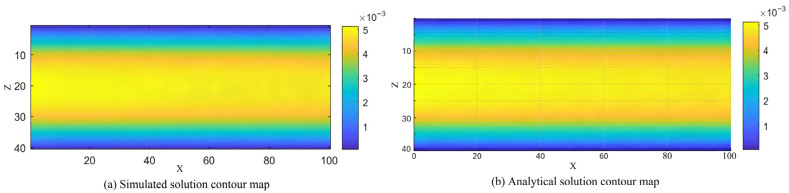
Comparison of Poiseuille flow contour maps.

Through a systematic analysis of the velocity contours, the development process of the flow within the channel can be clearly observed. Figure 16a presents the simulated velocity magnitude distribution (in units of 10^−3^) at time step t = 1720, where the x-direction denotes the flow direction and the y-direction represents the channel cross-section. The velocity values vary between 1 and 5 (i.e., 0.001 to 0.005), exhibiting typical laminar flow characteristics: near the inlet region, the velocity profile is not yet fully developed, and the boundary layer gradually forms; as the flow progresses downstream, the velocity distribution stabilizes, eventually forming a complete parabolic profile after the middle section of the channel. Figure 16b displays the theoretical analytical solution contour map, with velocity values ranging from 0 to 5 (i.e., 0 to 0.005), perfectly reproducing the ideal parabolic distribution of Poiseuille flow.

**Figure 17:**
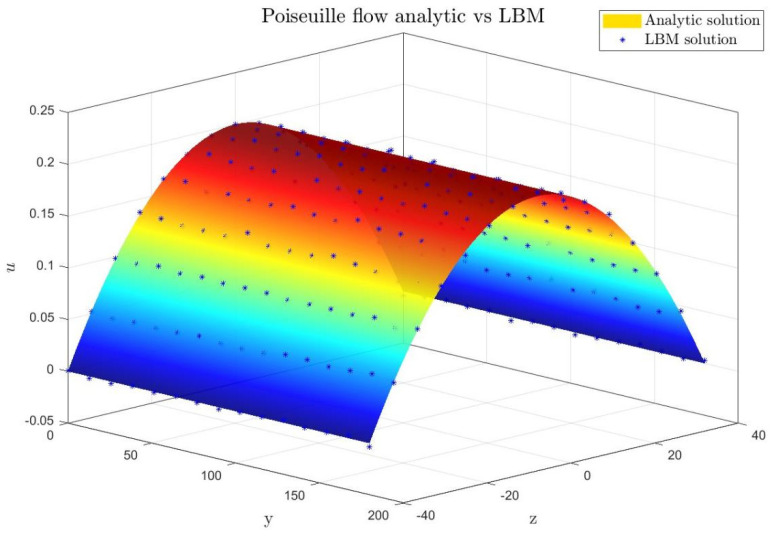
Fitting diagram of the simulated and analytical solutions for Poiseuille flow. (The blue surface represents the theoretical analytical solution, and the red scattered points represent the LBM simulation results).

To quantitatively evaluate the accuracy of the numerical simulation, this study systematically compares the velocity profiles obtained from the LBM simulation and the theoretical analytical solution in the fully developed region of the three-dimensional cylindrical channel Poiseuille flow. As shown in Figure 17, this plot uses the two spatial dimensions (y and z) of the channel cross-section as the horizontal axes and the normalized dimensionless velocity as the vertical axis. The velocity distributions from the theoretical analytical solution and the LBM simulation are presented as a surface and scatter points, respectively. The comparison results show a high degree of consistency across the entire channel cross-section: the velocity peaks in the central region of the channel (where y and z are near zero), then smoothly decreases radially, dropping to zero at the walls, exhibiting a complete and symmetric three-dimensional parabolic distribution. This distribution pattern aligns with the classical theoretical expectation for Poiseuille flow in a cylindrical channel, reflecting the flow’s symmetry and fully developed laminar characteristics. It is worth noting that the LBM simulation data closely fits the theoretical surface in all regions, including the near-wall boundary layer, with no significant deviations. This indicates that the D3Q27-MRT model and the associated boundary conditions employed in this study possess good numerical accuracy and stability when simulating three-dimensional pressure-driven flows and can accurately capture the essential flow behavior of Poiseuille flow. Therefore, this result not only validates the reliability of the LBM for simulating three-dimensional internal flow fields but also establishes a credible numerical foundation for its application to more complex flow systems.

**Figure 18:**
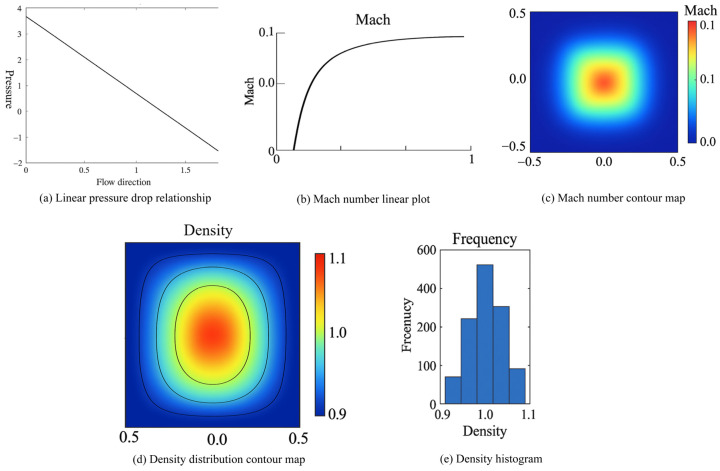
Collection of model validation and parameter analysis plots for three-dimensional cylindrical channel Poiseuille flow.

Based on the validation of the velocity field’s accuracy, this study further conducted a systematic analysis of the pressure distribution, Mach number, and density characteristics. Figure 18a shows the pressure distribution along the flow direction, where a well-defined linear decreasing trend from the inlet to the outlet is observed. This is entirely consistent with the characteristic constant pressure gradient in Poiseuille flow theory. Such a linear pressure distribution verifies the effectiveness of the Zou–He boundary condition in applying a minute pressure difference (Δ*p* = 0.000333), ensuring the accurate setting of the flow driving force. The Mach number analysis results are presented in Figure 18b,c. Figure 18b shows that the maximum Mach number within the flow domain remains below 0.1, which is significantly lower than the criterion threshold for incompressible flow (Ma < 0.3). The Mach number contour map in Figure 18c further indicates that the Mach number distribution is uniform throughout the entire computational domain, with no localized high-speed regions, satisfying the incompressibility assumption for lubricant flow in microscale seepage processes. This characteristic is crucial for accurately simulating low-velocity seepage behavior in porous media. The density field analysis results are shown in Figure 18d,e. The density distribution contour map reveals that the density variation amplitude across the entire flow domain is controlled within ±0.1%, exhibiting a smooth gradient distribution along the flow direction. The density histogram further demonstrates that the density values at all grid points are highly concentrated around the set value, with a standard deviation less than 0.05%. This proves that the model maintains good numerical stability and mass conservation characteristics even under minute pressure gradients.

#### 3.2.4. Grid Independence Study

To ensure that the subsequent seepage simulation results based on the real pore structure are independent of the grid size, a grid independence test was conducted in this study at a representative Reynolds number of Re = 400. The rationale for selecting Re = 400 is twofold: this Reynolds number lies in the critical transition region from laminar to transitional flow, where stable secondary vortices begin to appear within the cavity, effectively testing the model’s capability to capture complex vortex structures; furthermore, Re = 400 is widely used for validation in the literature and involves moderate computational cost.

**Figure 19:**
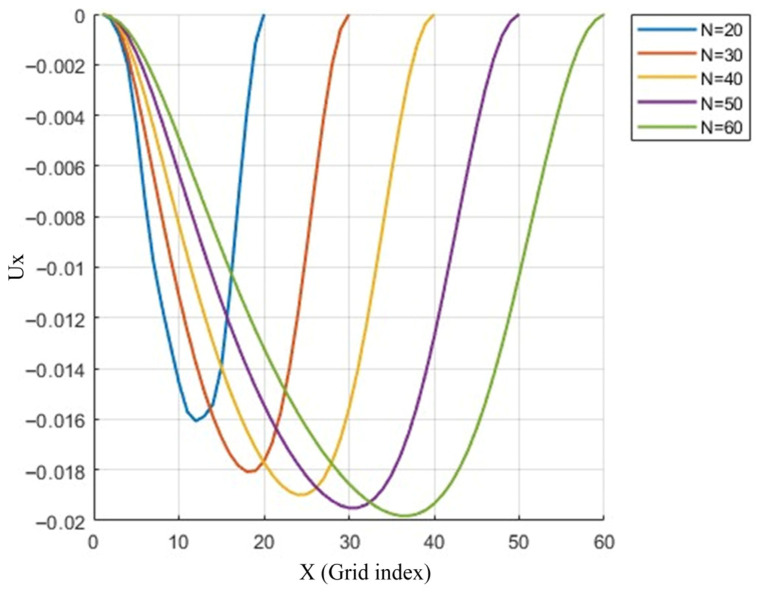
Grid independence study: Centerline Ux distribution for different grids at Re = 400.

Five uniform grid resolutions were employed: 20^3^, 30^3^, 40^3^, 50^3^, and 60^3^. Simulations were performed for lid-driven flow with the lid moving in the y-direction (the lid velocity corresponding to Re = 400). After the flow field reached a steady state, the distribution of the x-direction velocity component Ux along the centerline (y = Ny/2, z = Nz/2) was extracted, with the results shown in Figure 19. The negative values of Ux on the ordinate in the figure arise because, on this specific plane, the vortex structure induces fluid flow in the -x direction (the lid driving direction is +y; on the central plane y = Ny/2, the vortex causes the x-direction velocity to be negative in most regions). This phenomenon is entirely consistent with the physical characteristics of three-dimensional cavity flow.

As can be seen from Figure 19, the Ux curves gradually converge with grid refinement: the results for *n* = 50 and *n* = 60 are almost completely overlapping, indicating that the 50^3^ grid is sufficient to provide a grid-independent solution. Therefore, the adoption of the 50^3^ grid for subsequent simulations in this paper is both reasonable and economical, ensuring the accuracy and reliability of the numerical results.

## 4. Single-Phase Seepage Characteristics Analysis in Porous Polyimide

Based on the completed high-fidelity reconstruction of the three-dimensional pore structure (Section 2) and the systematic validation of the lattice Boltzmann model (Section 3), this section employs the established D3Q27-MRT model to simulate and analyze the single-phase seepage behavior of lubricating oil within porous polyimide under fixed geometric conditions. The study focuses on revealing the microscopic flow paths, velocity and pressure distributions, transport efficiency, and their key geometric controlling factors within the real, complex pore structure. Through mesoscopic numerical simulation, the aim is to elucidate the intrinsic physical mechanisms of seepage, thereby providing a quantitative basis for material performance optimization and lubrication system reliability design, and establishing a reliable physical benchmark for subsequent studies on the influence of surface properties (such as wettability, roughness). The analysis will follow the logical framework of “macroscopic transport characteristics—microscopic flow field structure—statistical quantitative description—structure-property correlation” to systematically explain its transport capacity, flow mechanism, distribution uniformity, and the controlling effect of pore structure on the flow.

### 4.1. Seepage Simulation Parameter Settings

Based on the reconstructed 3D digital model of porous polyimide from Section 2.3 (Figure 7c), its voxel data were directly used as the geometric input for LBM calculations. In the simulation, the lubricating oil was treated as an incompressible Newtonian fluid, with its physical parameters set according to typical aviation lubricants. A constant, small pressure difference was applied between the model’s inlet and outlet faces to simulate pressure-driven seepage. The magnitude of the pressure difference ensured the flow remained in the low Reynolds number laminar regime (Re << 1) and satisfied the incompressible condition of Mach number Ma < 0.1 (validated in Section 3.2.3). All solid walls were treated using the validated standard bounce-back scheme to simulate no-slip boundary conditions. The simulation employed the multiple-relaxation-time model (MRT-LBM, D3Q27), with relaxation parameters optimized to ensure numerical stability within the complex pore geometry. Iterative calculations were performed until the flow field reached a steady state (criterion: relative change in the global velocity field less than $10^{-6}$), providing a data foundation for subsequent visualization analysis and mechanism investigation.

To enable reproducible numerical simulations, this section supplements the key implementation parameters. The lattice spacing is directly determined by the CT scan resolution; i.e., Δx = 0.30383 μm. The lattice sound speed is taken as $c_{s}=1/\sqrt{3}$ in lattice units. The relationship between the fluid kinematic viscosity *ν* and the lattice viscosity is given by $\nu =c_{s}^{2}\left(\tau -0.5\right)\Delta t$, where *τ* is the relaxation time and Δ*t* is the time step. By matching the actual lubricating oil viscosity (reference value $\nu_{phys}$), the time step Δ*t* is determined from this relationship, thereby ensuring the physical consistency and numerical stability of the simulation. The inlet density is set to $\rho_{in}=1.01$ and the outlet density to $\rho_{out}=1.00$ (in lattice units), with the pressure gradient driving fluid seepage along the z-direction. The Mach number corresponding to this density difference satisfies Ma < 0.1, consistent with the incompressibility assumption. In the MRT model, the relaxation parameter related to viscous stress is set to $s_{\nu }=1/\tau$, while the remaining higher-order moment relaxation parameters are all set to 1.0 to ensure numerical stability. The convergence criterion is specifically defined as monitoring the change in the global velocity field every 100 iterations; convergence is considered achieved when $\epsilon =\max\left|u^{t}-u^{t-100}\right|/\max\left|u^{t}\right|<10^{-6}$. In practice, convergence was attained after approximately 2000 iterations. The parameter settings described above ensure the accuracy and reproducibility of the simulation results.

### 4.2. Evolution of Microscopic Flow Field Structure and Steady-State Characteristics

**Figure 20:**
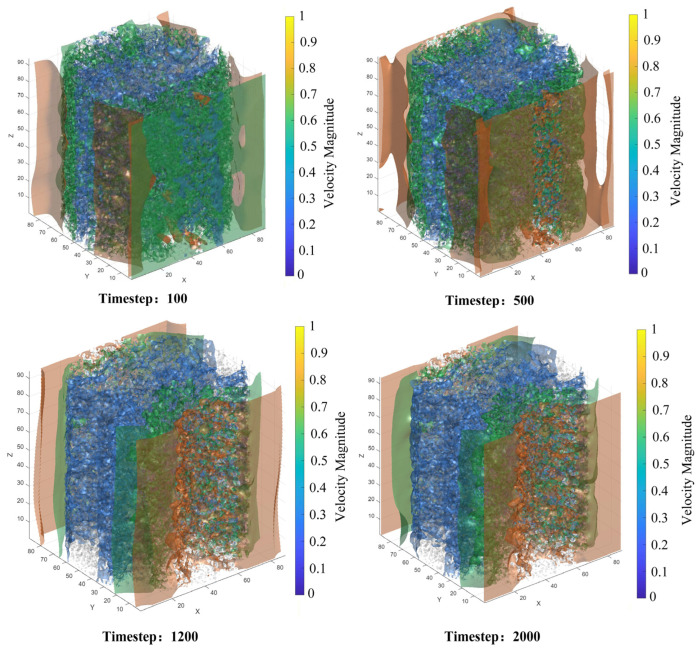
Three-dimensional isosurface distribution of velocity magnitude.

To deeply reveal the flow details of lubricating oil at the pore scale, three-dimensional visualization and quantitative analysis of the steady-state flow field were conducted in this study. Figure 20 presents the three-dimensional isosurface distribution of velocity magnitude, providing a global perspective for understanding the spatial structure of the flow field. In the figure, the X, Y, and Z axes correspond to the lattice coordinate directions, with units expressed in lattice units. The model grid used for seepage simulation has dimensions of 89 (X-direction) × 89 (Y-direction) × 94 (Z-direction) lattice units, with each lattice unit corresponding to the CT scan resolution of 0.3 μm. The color bar represents the velocity magnitude in lattice units, where red/warm colors indicate high-speed primary flow channels, and blue/cool colors indicate low-speed regions or near-wall boundary layers. It can be clearly observed from the figure that the high-speed regions represented by the red isosurfaces form a tortuous yet continuously interconnected primary channel network, constituting the “highway” for lubricating oil transport. The low-speed regions represented by the cyan/green isosurfaces occupy narrower secondary pore channels. Notably, a substantial portion of the pore space (particularly isolated pores) exhibits no generated isosurfaces, with velocities approaching zero. This indicates that the pore network actively participating in effective seepage is significantly smaller than the total pore space, intuitively revealing the primary screening effect of pore structure on flow pathways.

**Figure 21:**
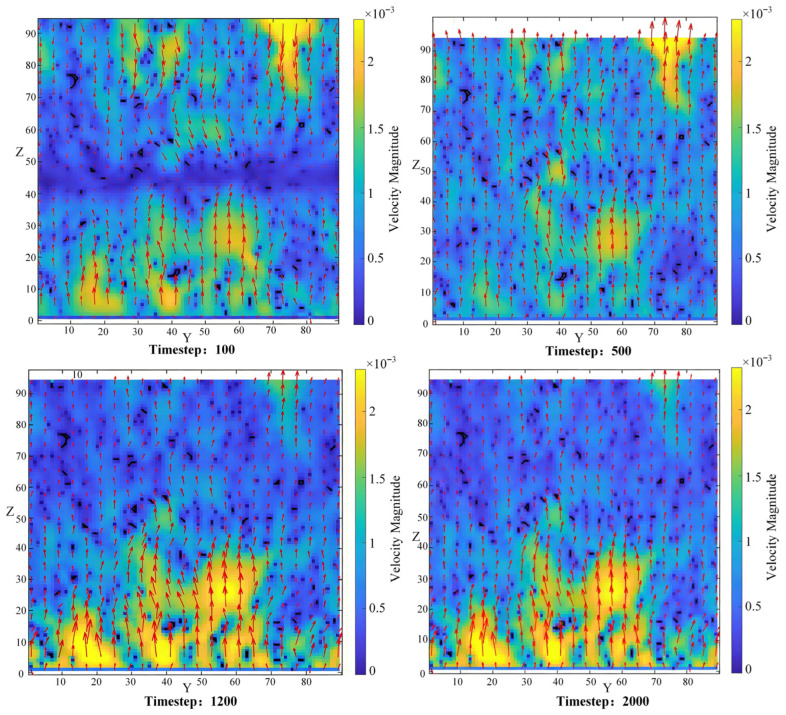
Velocity vector diagram (YZ plane, X = 45).

To trace the complete evolution of the flow from the initial transient state to the fully steady state, Figure 21 presents contour maps of velocity magnitude distribution on the YZ plane (X = 45 cross-section) at different time steps. In the figure, the horizontal axis represents the Y-direction, and the vertical axis represents the Z-direction, with units in lattice units. The cross-section is located at the 45th lattice point in the X-direction of the model, corresponding to the middle position of the model. The color bar represents the velocity magnitude in lattice units, transitioning from blue (low velocity) to yellow (high velocity).

At the initial time step (Timestep = 100), the velocity distribution is chaotic, with high-velocity regions scattered sporadically, indicating that the fluid is exploring and filling the pore network, and the inlet effect is significant. As the iteration proceeds (Timestep = 500, 1200), the flow field gradually self-organizes: the velocity along the primary flow paths increases significantly, forming continuous high-velocity channels that clearly outline the specific pathways of the fluid as it bypasses the solid skeleton and flows through the throats. By Timestep = 2000, the flow field has reached a fully steady state, with the velocity distribution in the figure remaining unchanged. The high-velocity channels stably permeate the pore network, exhibiting a noticeable acceleration effect (local velocity increase) particularly at narrow throats. This vividly demonstrates the precise spatial coupling relationship between the steady-state flow field and the solid skeleton.

**Figure 22:**
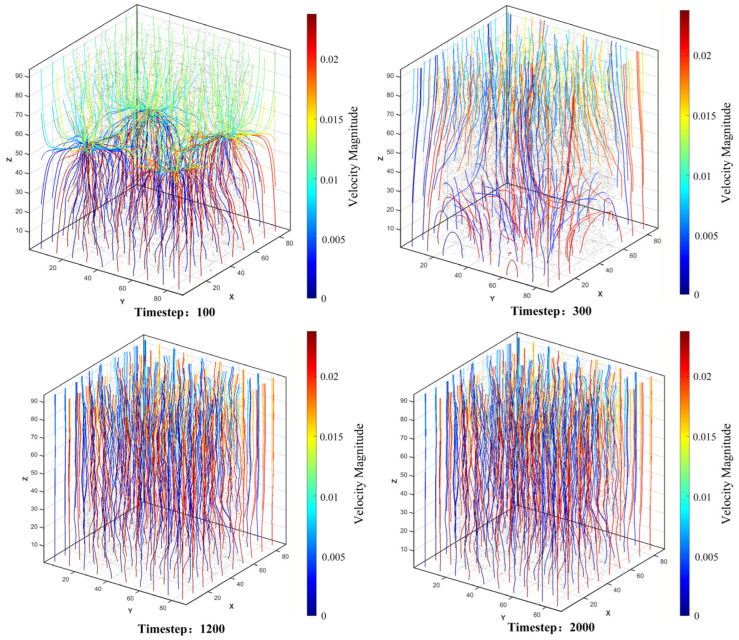
Streamline plot.

To further understand the flow paths from the perspective of fluid particle trajectories, Figure 22 provides three-dimensional streamline diagrams based on the Lagrangian viewpoint, illustrating the streamline evolution process at time steps 100, 300, 1200, and 2000. At Timestep = 100, the streamlines are chaotic and disorganized, with no clear paths yet formed. By Timestep = 300, some streamlines begin to converge, initially outlining the embryonic form of preferential channels. As the flow develops (Timestep = 1200), the preferential paths become further clarified. Finally, at Timestep = 2000, the streamlines are completely stabilized, forming several primary flow channels that penetrate the pore network. The streamlines are highly tortuous in three-dimensional space, frequently bifurcating and merging. At the macroscopic level, this forms several preferential transport paths, while at the microscopic level, it reveals a periodic motion pattern of “acceleration-expansion-deceleration” as the fluid navigates the alternating pore-throat structures. Some streamlines terminate in blind pores, creating flow dead zones. These zones corroborate the missing regions observed in the velocity isosurfaces, collectively confirming the heterogeneity of the pore space in terms of transport functionality.

### 4.3. Mechanism of Pore Structure Regulation on Seepage

The aforementioned streamline plots, isosurfaces, and vector diagrams illustrate the macroscopic patterns and evolutionary process of seepage. However, their formation originates from the microscopic pore structure. This section will delve into how pore topology precisely regulates the flow field by analyzing the quantitative distributions of pressure and velocity scalar fields.

**Figure 23:**
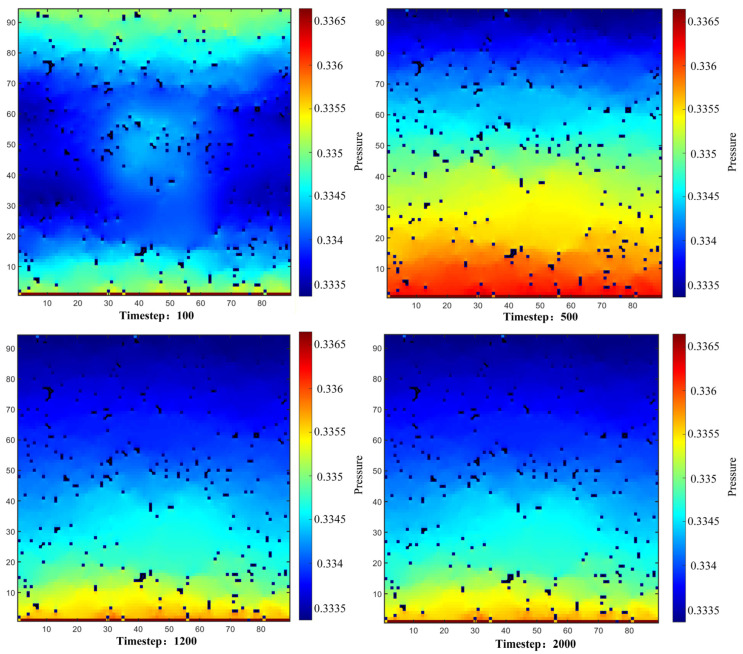
Pressure distribution contour map. (The colors represent pressure magnitude, with red indicating high-pressure regions and blue indicating low-pressure regions. The black square areas represent the solid skeleton of the porous polyimide, while the white/colored areas represent the fluid within the pore spaces).

Pressure contour maps (as shown in Figure 23) reveal the spatial and temporal distribution characteristics of the pressure field driving the flow. The figure displays the same four time steps (100, 500, 1200, 2000) selected for Figure 20 and Figure 21, facilitating a comparative analysis of the evolutionary relationship between the velocity field and the pressure field. Overall, the pressure exhibits a quasi-linear decline along the flow direction, aligning with the macroscopic manifestation of Darcy’s law. However, during the evolution process (e.g., between Timestep = 500 and 1200), local anomalies in the pressure gradient appear. This phenomenon directly points to the presence of topological mutations in the pore space at these locations, such as sharp contractions in throats or pronounced bends in flow paths, leading to a sudden increase in local flow resistance and thereby constituting a detectable “signal” in the macroscopic pressure field. By Timestep = 2000, the pressure field has reached stability, yet the local anomalies persist, underscoring the persistent regulatory role of the pore structure on the pressure field.

**Figure 24:**
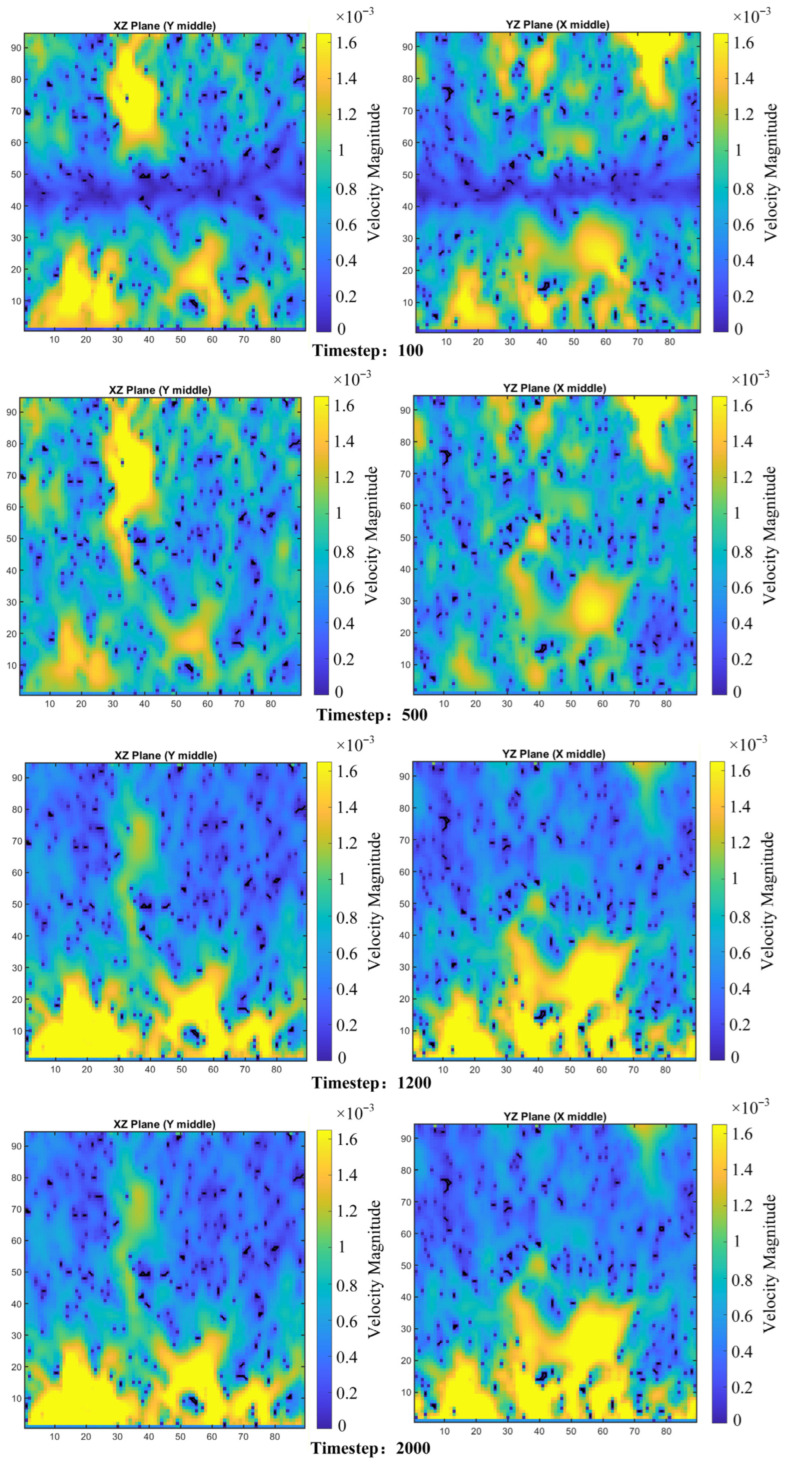
Velocity contour map.

Velocity contour maps (as shown in Figure 24) provide a continuous quantitative distribution of velocity magnitude on a representative cross-section (XZ plane at the mid-Y location), with the evolution process illustrated at time steps 100, 500, 1200, and 2000. The contour maps clearly demonstrate that high-velocity regions (warm colors) are strictly confined to the central areas of well-connected large pore channels, while an extremely thin low-velocity boundary layer (cool colors) exists near the pore walls. As the time steps progress, the high-velocity channels gradually form and stabilize; by Timestep = 2000, the velocity distribution no longer changes. More importantly, the “pattern” of the velocity distribution exhibits an exceptionally high degree of matching with the pore geometry—every increase or decrease in flow velocity corresponds to an expansion or contraction of the flow channel cross-section. This strong correlation demonstrates that the local flow velocity is primarily constrained by the instantaneous geometric dimensions of the specific throat through which it flows.

**Figure 25:**
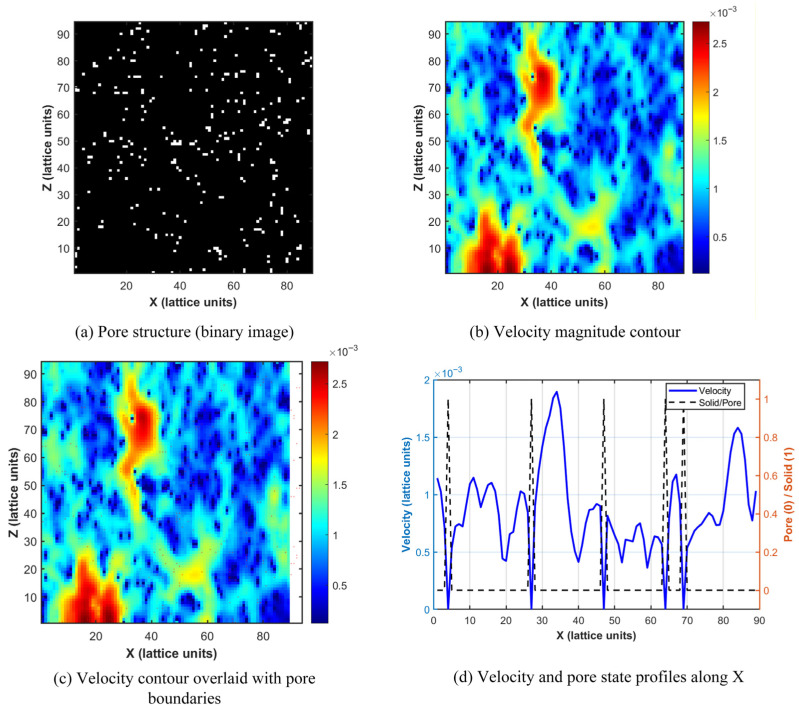
Correlation analysis between velocity distribution and pore geometry. (XZ plane, Y middle section, Timestep = 2000).

To further quantitatively verify the intrinsic correlation between velocity distribution and pore geometry, Figure 25 presents the pore structure, velocity contour map, and their overlay on the same cross-section (XZ plane at the mid-Y location) under fully steady-state conditions (Timestep = 2000). Figure 25a shows the binary image of the pore structure, where black areas represent pores and white areas represent the solid skeleton, providing a geometric reference for subsequent comparison. Figure 25b displays the corresponding velocity magnitude contour map, with the color mapping consistent with Figure 24, clearly illustrating the heterogeneous distribution of velocity within the pore space. Figure 25c overlays the pore boundaries onto the velocity contour map, intuitively revealing that high-velocity regions (warm colors) are strictly confined within the pores, and the velocity gradient variations closely align with the pore boundary morphology. Figure 25d extracts the velocity profile (blue curve) and pore state (black dashed line) along the path indicated by the white dashed line in Figure 25c (at a fixed Z-coordinate). Quantitative comparison demonstrates that the velocity peaks precisely correspond to the narrowest throat constrictions, while the velocity troughs correspond to the widest pore bodies. Furthermore, the magnitude of velocity variation is positively correlated with the degree of cross-sectional change. This result directly proves that the local flow velocity is primarily constrained by the instantaneous geometric dimensions of the pores, providing intuitive and quantitative evidence for the mesoscopic mechanism where “structure governs flow.”

**Figure 26:**
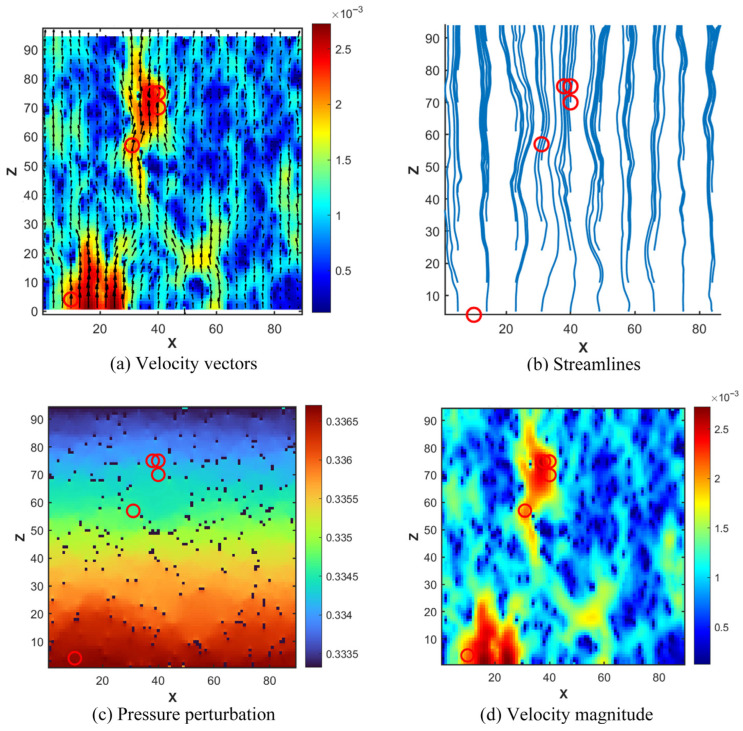
Comprehensive visualization of the steady-state flow field with multi-physical quantities.

To further comprehensively demonstrate the flow characteristics at large throats, Figure 26 presents multiple visualizations of the flow field on the same cross-section (XZ plane, Y middle section) at the steady-state time step (Timestep = 2000). The approximate locations of large throats, identified based on the velocity threshold corresponding to the large throat size range determined by mercury intrusion porosimetry, are marked with red circles. Figure 26a shows the velocity vector diagram, where vectors at the large throats are dense and directionally concentrated, indicating fluid acceleration through these constrictions. The streamline diagram in Figure 26b reveals that large throats serve as preferential pathways where streamlines converge. Figure 26c presents the pressure perturbation map (obtained by subtracting the streamwise-averaged pressure), where the black squares represent the solid skeleton. The locations of large throats, marked by red circles, clearly reveal the local pressure drops (blue regions) resulting from increased flow velocity at these large throats. This local pressure anomaly, which would otherwise be indiscernible in the original pressure contour, is effectively highlighted through the perturbation processing. The velocity contour map in Figure 26d once again confirms the high-velocity characteristics at large throats. Comparative analysis across these figures demonstrates that large throats are not merely geometrically wide channels but also serve as hydrodynamic high-velocity pathways and pressure drop centers. Their locations closely align with the size threshold defined by mercury intrusion porosimetry, thereby microscopically validating the mesoscopic mechanism that “structure governs flow.”

Integrating the above analysis, the steady-state seepage behavior of lubricating oil in porous polyimide—manifested as the localization of preferential pathways, the formation of dead zones, and fluctuations in the pressure field—is not a random phenomenon but rather an inevitable manifestation and precise regulatory outcome of the micro-scale pore structure (throat size distribution, local connectivity, and tortuosity) underpinned by fluid mechanics principles. Specifically, the throat size distribution, by controlling local flow resistance, directly governs the spatial allocation of velocity and the global flux distribution. Visualization evidence confirms that high-velocity paths strictly correspond to large throats, and a minority of these large throats dominate the vast majority of transport. Connectivity between pores shapes the topological structure of the flow network at a higher dimensional level: highly connected nodes act as “hubs” for streamline convergence and divergence, dominating flow redistribution, whereas poorly connected regions tend to form low-velocity zones or flow dead zones. High flow resistance and energy dissipation induced by locally high tortuosity or narrow throats manifest as local anomalies in the pressure gradient within the macroscopic pressure field.

Through a combination of visualization and quantitative analysis, this study profoundly reveals the micro-mechanical mechanism by which “structure governs flow,” delineating the regulatory pathways through which pore topological characteristics influence seepage behavior. This understanding provides a direct theoretical basis and identifies clear regulatory targets for optimizing the lubricant transport performance of porous polyimide materials through microstructural design.

## 5. Conclusions and Outlook

This study systematically conducted mesoscale research on the three-dimensional digital model reconstruction and single-phase seepage characteristics of porous polyimide by integrating micro-focus CT scanning, image processing, three-dimensional reconstruction, and the lattice Boltzmann method. The main conclusions are as follows:

1. A high-fidelity three-dimensional digital model of porous polyimide was successfully constructed. Using micro-focus CT scanning and image processing techniques such as non-local means filtering and threshold segmentation, precise conversion from a real sample to a digitized pore structure was achieved. The constructed model and the extracted pore network model not only intuitively reproduce the complex, interconnected three-dimensional pore space within the material but also quantify its key geometric and topological parameters (e.g., porosity, throat size distribution, coordination number). This provides a realistic and reliable geometric foundation for subsequent seepage numerical simulations.
2. A D3Q27-MRT lattice Boltzmann model suitable for seepage simulation in complex porous media was established and validated. Through systematic validation using two classic benchmark cases—lid-driven cavity flow and Poiseuille flow—it was confirmed that the adopted model and boundary conditions possess good numerical accuracy, stability, and the capability to handle complex solid wall boundaries when simulating different flow types. This lays a credible numerical computational foundation for conducting seepage simulations within real pore geometries.
3. The microscopic mechanisms and structural control laws of single-phase lubricant seepage in porous polyimide were revealed. Simulation results indicate that the seepage process exhibits significant heterogeneity: the flow spontaneously forms several high-speed “preferential paths,” while most of the pore space contributes minimally to macroscopic transport. In-depth analysis found that pore topology is the decisive factor regulating seepage behavior: the throat size distribution directly controls the spatial velocity allocation and global flow rate, demonstrating the rule that “a few large throats dominate most of the transport”; the connectivity (coordination number) between pores determines the topological shape of the flow network, governing fluid convergence, distribution, and the formation of dead zones; locally high tortuosity or narrow throats cause a sharp increase in flow resistance, manifesting as obvious gradient anomalies on the macroscopic pressure field. This quantitative correlation mechanism clarifies that the steady-state seepage field is the inevitable result of fluid obeying mechanical laws under the constraints of the inherent pore structure.
4. A complete research paradigm linking “structure-simulation-performance” was established. This study connects the entire process from experimental observation (CT scanning) to structural digitization (3D reconstruction), then to physical field numerical simulation (LBM) and mechanistic analysis, forming a complete technical chain. This paradigm not only provides direct theoretical basis and predictive tools for studying the seepage characteristics of porous polyimide, but its methodological framework can also be extended to the analysis and design of transport performance in other porous functional materials.

In summary, this study, from a mesoscopic scale, reveals the fundamental law that lubricant seepage behavior in porous polyimide is precisely regulated by its microscopic pore structure, deepening the understanding of the material’s oil storage and supply mechanism. The research results provide clear theoretical guidance and key design parameters for enhancing the performance of porous polyimide lubricating materials by optimizing the pore structure (e.g., controlling throat size, improving connectivity). Future work can build upon this foundation to further investigate more complex seepage behaviors, considering factors such as surface wettability, multiphase flow, and dynamic cyclic loading conditions.

## Figures and Tables

**Table 1:** Micro-CT scanning parameters.

Parameter	Value
Model	Rmct-4000
Voltage	200 kV
Current	200 μA
Power	200 W
Exposure Time	3 s
Magnification	40×
Binning Mode	2 × 2
Reconstruction Algorithm	Filtered Back Projection (FBP)
Voxel Size	0.30383 μm (isotropic)
Number of Scan Slices	959
Pixel Matrix	2048 × 2048 pixels
Detector Type	Flat-panel digital detector

**Table 2:** Pore structure parameters of porous polyimide.

Parameter	Value	Unit
Porosity	8.55	%
Specific surface area	23.66	m^2^/g
Average throat diameter (≥300 nm)	624.7	nm
Large throat threshold (μ+σ)	5383.2	nm

**Table 3:** Discrete velocity vectors and weighting coefficients for the D3Q27 model.

Index i	$\mathrm{Velocity}\;\mathrm{Vector}\;e_{i}=(e_{ix},e_{iy},e_{iz})$	$\mathrm{Squared}\;\mathrm{Velocity}\;\mathrm{Magnitude}\;{\left|e_{i}\right|}^{2}$	$\mathrm{Weighting}\;\mathrm{Coefficient}\;w_{i}$
0	(0, 0, 0)	0	8/27
1	(1, 0, 0)	1	2/27
2	(−1, 0, 0)	1	2/27
3	(0, 1, 0)	1	2/27
4	(0, −1, 0)	1	2/27
5	(0, 0, 1)	1	2/27
6	(0, 0, −1)	1	2/27
7	(1, 1, 0)	2	1/54
8	(−1, 1, 0)	2	1/54
9	(1, −1, 0)	2	1/54
10	(−1, −1, 0)	2	1/54
11	(1, 0, 1)	2	1/54
12	(−1, 0, 1)	2	1/54
13	(1, 0, −1)	2	1/54
14	(−1, 0, −1)	2	1/54
15	(0, 1, 1)	2	1/54
16	(0, −1, 1)	2	1/54
17	(0, 1, −1)	2	1/54
18	(0, −1, −1)	2	1/54
19	(1, 1, 1)	3	1/216
20	(−1, 1, 1)	3	1/216
21	(1, −1, 1)	3	1/216
22	(−1, −1, 1)	3	1/216
23	(1, 1, −1)	3	1/216
24	(−1, 1, −1)	3	1/216
25	(1, −1, −1)	3	1/216
26	(−1, −1, −1)	3	1/216

## Data Availability

The original contributions presented in this study are included in the article. Further inquiries can be directed to the corresponding author.
